# Supplementary material for: What are the important morbidities associated with paediatric cardiac surgery? A mixed methods study
Source: BMJ Open. 2019 Sep 9;9(9):e028533. doi: 10.1136/bmjopen-2018-028533 (PMC6738689; doi:10.1136/bmjopen-2018-028533)
Supplement: Supplementary data [file bmjopen-2018-028533supp001.pdf]

Appendix 1 from Brown KL, Pagel C, Brimmell R*, et al.* Definition of important early morbidities related to paediatric cardiac surgery. *Cardiol Young*. 2017;**27**:747-56 doi: 10.1017/S1047951116001256 [published Online First: 2016/09/30].

Morbidity	Timescale for identification	Definition	Measurement protocol (if additional to definition)	Minimum treatment protocol
**Acute Neurological Event**	Includes neurological morbidities that, based on best clinical judgement, arose as *new* findings around the time of surgery that were detected within the same hospitalisation as the surgery. It is recognised that in certain circumstances such as where a child is very sick on life support, pre procedure assessment is challenging, in these circumstances as full an evaluation as possible to be completed, incorporating serial assessments over time.	Neurological events including: seizure, abnormal movement (includes choreiform or athetoid), focal neurological deficit (includes hemiplegia and monoplegia), intracranial haemorrhage, stroke, brain death, Reversible Ischaemic Neurological Dysfunction, Hypoxic Ischaemic Encephalopathy, spinal cord ischaemia, basal ganglia damage or brain stem injury (includes abnormal cough or gag reflex). (1)	Includes new abnormality in any of the following:<br>- Electroencephalogram.<br>- Brain scan (either computerised tomography or magnetic resonance).<br>- Clinical evaluation<br>(Seizures or movement disorder, focal neurological signs, generalised neurological signs, altered conscious level including even brain death.)	The treatment protocol is variable depending on the type of neuro-morbidity.<br>Specialist consultation with a neurologist, a full evaluation of any brain injury and neuro-developmental follow up would be a minimum.

**Unplanned reoperation or re-intervention**	Unplanned re-interventions are procedures outside the expected patient pathway, which may be undertaken at any time from the start of the post-operative admission up until 30 days following the primary operation. Additional procedures or revisions undertaken within the primary trip to the operating theatre (incorporating return onto cardiopulmonary bypass) are not included in the definition of re-operation.	Unplanned re-interventions include procedures that were not intended during the planning phase, follow an initial primary cardiac surgery and result in "substantive alteration to heart" incorporating cardiac bypass, cardiac non bypass, pacemaker placement, interventional catheterisations and also diaphragm plications (which are not related to the heart itself). The definition does not include support or other non-cardiac surgery procedures.	Unplanned return to the operating room or cardiac catheter laboratory within 30 days<br>(Excludes interventional catheters that were planned pre-operatively; excluding delayed chest closure, excluding procedures for bleeding)<br>(Includes diaphragm plication and insertion of pace maker for surgically acquired arrhythmia).	Not applicable. The minimal assessment is cardiovascular evaluation of the repair with echocardiography and tolerance of weaning from life supports.
**Feeding problems**	A diagnosis of post-operative feeding problems should be considered during recovery after surgery and prior to discharge from the specialist centre either to home or to secondary care if	A child may fail to feed normally following paediatric cardiac surgery for a range of reasons including gastro-oesophageal reflux, vocal cord paralysis, oral-motor dysfunction, oral aversion, and neurologic impairment. (2) If for any of these reasons a child is not able to orally	The requirement for any feeding support.<br>Includes via the intravenous route or via an enteral tube.	Treatment includes assessment by the dietician, speech and language therapist and of the patient's weight. Progress with

	the child is unable to feed normally. The goal is detection of feeding problems which are new post-surgery, and it is recognised that this may be challenging where a child was not fed pre-operatively for cardiac reasons since feeding ability will not have been assessed objectively.	feed or completely orally feed and is tube dependent at discharge from the tertiary centre or at 30 days (if he or she is otherwise clinically stable enough to feed at that time point), then a post-operative feeding problem will be diagnosed.	Excludes feeding support that was present to treat a primary problem diagnosed before the surgery, feeding support related to an episode of necrotising enterocolitis, and feeding support because the child dislikes a special diet.	feeding should be monitored by the clinical care team responsible at each stage of the journey.
**Need for renal replacement therapy**	Includes renal replacement therapy when initiated as a new support at any time from the start of the post-operative admission to ICU up until 30 days following the primary operation.	The child requires renal replacement therapy (peritoneal dialysis or haemofiltration) for renal failure (oligo-anuria of less than 0.5 ml/kg/hour and elevated creatinine level for age) and or fluid overload. In patients where renal support is required alongside extracorporeal life support, the primary morbidity is viewed as extracorporeal life support.	The measurement protocol is simply the presence of (new) renal support. (Excludes renal support on extracorporeal life support). Data on renal biochemistry and urine output will be collected.	Instigation of effective renal replacement therapy.<br>If recovery of kidney function does not occur within 3 to 5 weeks then consultation with paediatric renal

				physician is required.
**Major adverse cardiac events or never events**	Events within this morbidity may be identified during the tertiary hospital stay (either ward or ICU) following the primary surgery.	These morbidity includes:<br><br>- Cardiac arrest, where the child receives any chest compressions or defibrillation.<br><br>- Chest re-opening on the ICU or ward for any reason.<br><br>- Major haemorrhage in the ICU following surgery.<br><br>- A 'Never Event' applicable to paediatric cardiac surgery as selected from the 'Never Events' list published for NHS for 2015 (3)<br><br>(Including wrong site or wrong patient surgery, wrong prosthesis surgery, retained foreign object post procedure, wrong route administration of medication, transfusion or transplantation of main red cell group incompatible blood components or organs, misplaced naso-gastric or oro-gastric tubes,	Major haemorrhage is defined as bleeding > 10ml/kg/hr on ICU for 2 consecutive hours.<br><br>A 'Never Event' includes the events listed plus harm to the patient, for example: if a naso-gastric tube is misplaced, detected and removed in a timely manner before any harm is done then this is not a 'Never Event'. Conversely, if the misplaced naso-gastric tube is not noted, and feed is given into the bronchus, then this is a 'Never Event'.	All events will results in immediate treatment as part of current practice.

		- Tissue injury to limb or vital organ such as perforated viscus or ischaemic limb injury.		
**Extracorporeal life support**	Extracorporeal life support following surgery and before discharge from the tertiary hospital, including the rare cases when a child was on extracorporeal life support before surgery.	This morbidity is defined by the presence of an extracorporeal life support system connected to the patient following the operation, whether it was placed in the operating theatre or in the intensive care unit, and whether the indication was cardiac arrest, low cardiac output state, poor cardiac function, arrhythmia, residual or recurrent cardiac lesion, pulmonary including pulmonary hypertension or sepsis.	It is recognised that children on extracorporeal life support following paediatric cardiac surgery have high rates of other complications including renal support, bleeding, sepsis, sternal reopening, and cardiac arrest. (4) Where such complications arise as part of extracorporeal life support, the morbidity is defined as extracorporeal life support.	The morbidity is in fact a treatment modality offered so this is not applicable. Centres offering extracorporeal life support follow protocols based on those provided by the extracorporeal life support organisation. [24]
**Necrotising enterocolitis**	Necrotising enterocolitis as a new diagnosis from after surgery until discharge from the tertiary hospital.	Necrotising enterocolitis class 1a or 1b, (5) which incorporates babies with systemic signs of inflammation and abdominal clinical signs such as distension or larger than normal gastric aspirates or mild rectal	Data in respect of systemic clinical signs, intestinal signs and radiology will be collected, as well as the	Consultation with general surgery and further management in respect of antibiotics,

		bleeding but no radiological changes are included, if a general surgery specialist has seen the child and commenced a course of intravenous antibiotics and parenteral nutrition for five to seven days. Cases of severe necrotising enterocolitis with radiological signs systemic instability and bowel perforation are also included.	treatments deployed, thus enabling the necrotising enterocolitis diagnosis to be graded between 1a and 3b. [25]	nutrition, radiological investigation and surgical intervention.
**Surgical site infection and bloodstream infection**	Surgical site and blood stream infections diagnosed within the hospital admission following surgery or following readmission to the same unit during post-operative recovery, where the treating clinical team assesses the infection to be linked to the recent operation. It is noted that mediastinitis may be detected more than 30 days after cardiac surgery (6) hence this time cut off is not applicable.	Deep surgical site Infection and/or mediastinitis includes any infection of an incised wound that undergoes any re intervention by a surgeon (such as opening of the wound, vacuum dressing), mediastinitis and false aneurysm, independent of culture positivity. [23]<br><br>Blood stream infection includes both catheter related and non-catheter related. Cases have systemic signs of infection, a positive culture not judged to be a contaminant, and in the case of line related a catheter in place with positive cultures from the line or from the line tip when removed.	Deep surgical site infection excludes superficial site infection managed without a surgeon's reoperation by conventional nurse dressing only, even if the wound heals by secondary intention.	The minimum treatment protocol consists of antibiotics based on organism and sensitivities, and where relevant the removal of the line. Surgical intervention may be required for deep surgical site and in some cases of endocarditis. Both conditions require

		Endocarditis based on clinical, imaging or culture evidence judged to be diagnostic of endothelial/endocardial infection and its sequelae cardiac or extra-cardiac.		prolonged antibiotic therapy.
**Prolonged pleural effusion or chylothorax**	Prolonged pleural effusion is a post procedural effusion with duration greater than ten days. Chylothorax is diagnosed from after surgery until discharge from the tertiary hospital.	Either a chylous pleural effusion or significant chylous pericardial effusion or significant chylous ascites or a prolonged non-chylous effusion that necessitates thoracic drainage at least ten days following index cardiac surgery.	Chylous effusions are characterised by milky appearance and a pleural fluid white blood cell count of greater than 1000 cells/µl with lymphocytes greater than 80%. (7) If the child is on normal feeds the triglyceride level in the pleural fluid will be > 1.1 mmol/L or the ratio between the pleural triglyceride level and the serum triglyceride level will exceed 1.	Diet consisting of medium chain triglycerides or low fat for chylothorax. On a patient-by-patient basis other treatments include parenteral nutrition, octreotide infusion, intervention for venous obstruction thoracic duct ligation, and pleuradhesis.

1. Dominguez TE, Wernovsky G, Gaynor JW. Cause and prevention of central nervous system injury in neonates undergoing cardiac surgery. *Seminars in thoracic and cardiovascular surgery*. 2007;**19**:269-77 doi: 10.1053/j.semtcvs.2007.07.005 [published Online First: 2007/11/07].
2. Medoff-Cooper B, Naim M, Torowicz D, Mott A. Feeding, growth, and nutrition in children with congenitally malformed hearts. *Cardiol Young*. 2010;**20 Suppl 3**:149-53 doi: 10.1017/S1047951110001228 [published Online First: 2010/11/20].
3. NHS. Never-events. In: England N, ed. *Patient Safety*. London: NHS England 2015:Patient Safety Guideline.
4. Chaturvedi RR, Macrae D, Brown KL*, et al.* Cardiac ECMO for biventricular hearts after paediatric open heart surgery. *Heart*. 2004;**90**:545-51 Online First: 2004/04/16].
5. McElhinney DB, Hedrick HL, Bush DM*, et al.* Necrotizing enterocolitis in neonates with congenital heart disease: risk factors and outcomes. *Pediatrics*. 2000;**106**:1080-7 Online First: 2000/11/04].
6. Sohn AH, Schwartz JM, Yang KY, Jarvis WR, Guglielmo BJ, Weintrub PS. Risk factors and risk adjustment for surgical site infections in pediatric cardiothoracic surgery patients. *Am J Infect Control*. 2010;**38**:706-10 doi: 10.1016/j.ajic.2010.03.009 [published Online First: 2010/07/08].
7. Zuluaga MT. Chylothorax after surgery for congenital heart disease. *Curr Opin Pediatr*. 2012;**24**:291-4 doi: 10.1097/MOP.0b013e3283534b7f [published Online First: 2012/04/14].
