# Supplementary material for: What are the important morbidities associated with paediatric cardiac surgery? A mixed methods study
Source: BMJ Open. 2019 Sep 9;9(9):e028533. doi: 10.1136/bmjopen-2018-028533 (PMC6738689; doi:10.1136/bmjopen-2018-028533)
Supplement: Author's manuscript [file bmjopen-2018-028533.draft_revisions.pdf]

BMJ Open

BMJ Open is committed to open peer review. As part of this commitment we make the peer review history of every article we publish publicly available.

When an article is published we post the peer reviewers' comments and the authors' responses online. We also post the versions of the paper that were used during peer review. These are the versions that the peer review comments apply to.

The versions of the paper that follow are the versions that were submitted during the peer review process. They are not the versions of record or the final published versions. They should not be cited or distributed as the published version of this manuscript.

BMJ Open is an open access journal and the full, final, typeset and author-corrected version of record of the manuscript is available on our site with no access controls, subscription charges or pay-per-view fees (http://bmjopen.bmj.com).

If you have any questions on BMJ Open's open peer review process please email info.bmjopen@bmj.com

BMJ Open

What are the important morbidities associated with paediatric cardiac surgery? : A mixed methods study

Journal:	*BMJ Open*
Manuscript ID	bmjopen-2018-028533
Article Type:	Research
Date Submitted by the Author:	13-Dec-2018
Complete List of Authors:	Brown, Katherine; Great Ormond Street Hospital for Children, Cardiorespiratory Division<br>Pagel, Christina; UCL<br>Ridout, Deborah; UCL, Institute of Child Health<br>Wray, Jo; Great Ormond Street Hospital for Children, Cardiorespiratory Division<br>Anderson, David; Evelina London<br>Barron, David; Birmingham Women's and Children's NHS Foundation Trust<br>Cassidy, Jane; Birmingham Women's and Children's NHS Foundation Trust<br>Davis, Peter; Bristol Royal Hospital for ChilDr.en, Paediatric Intensive Care<br>Hudson, Emma; Health Economics, University College London<br>Jones, Alison; Birmingham Women's and Children's NHS Foundation Trust<br>Mclean, Andrew; Royal Hospital for Children, Congenital Heart Surgery<br>Morris, Stephen; University College London, Department of Applied Health Research<br>Rodrigues, Warren; Royal Hospital for Children Glasgow<br>Sheehan, Karen; Bristol Royal Hospital for Children<br>Stoica, Serban ; University Hospitals Bristol NHS Foundation Trust<br>Tibby, Shane; Evelina Childrens Hospital, Paediatric Intensive Care<br>Witter, Thomas; Evelina London Children's Healthcare<br>Tsang, Victor; Great Ormond Street Hospital for Children, Cardiorespiratory Division
Keywords:	PAEDIATRICS, Cardiac surgery < SURGERY, complications, quality of life

SCHOLARONE™
Manuscripts

What are the important morbidities associated with paediatric cardiac surgery? : A mixed methods study

Authors

Katherine L Brown MD[1], Christina Pagel PHD[2], Deborah Ridout MSc[3], Jo Wray PHD[1], David Anderson FRCS[4], David J Barron MD[5], Jane Cassidy MRCP[5], Peter Davis FRCPCH[6], Emma Hudson MSc[8], Alison Jones MSc[5], Andrew McLean FRCS[10], Steve Morris PHD[8], Warren Rodrigues MRCPCH[10], Karen Sheehan MSc[6], Serban Stoica FRCS[6], Shane Tibby MRCP[4], Thomas Witter RN[4], Victor T Tsang FRCS[1]

[1]Charles West Division, Great Ormond Street Hospital NHS Foundation Trust, Great Ormond Street, London

[2]Clinical Operational Research Unit, University College London, London

[3]Population, Policy and Practice Programme, UCL Great Ormond Street Institute of Child Health, London

[4]Department Paediatric Cardiology and Cardiac Surgery, Evelina London Children's Hospital, London

[5]Department of Intensive care and Paediatric Cardiac Surgery, Birmingham Children's Hospital, Birmingham

[6]Paediatric Intensive Care Unit and Department Paediatric Cardiac Surgery, Bristol Royal Hospital for Children, Bristol

[7]Paediatric Cardiology Department, Royal Brompton and Harefield NHS Foundation Trust, London

[8]Health Economics, Department of Applied Health Research, UCL

[9]Community Child Health, UCL Great Ormond Street Institute of Child Health, London

[10]Department of Intensive care, Royal Hospital for Children, Glasgow

Corresponding author

Katherine L Brown Great Ormond Street Hospital NHS Foundation Trust, Great Ormond Street, London WC1N 3JH

Katherine.brown@gosh.nhs.uk

Phone 0207 405 9200 extension 8016 or 8180

Fax 0207 829 8673

For peer review only - http://bmjopen.bmj.com/site/about/guidelines.xhtml

Keywords – Paediatrics, cardiac surgery, outcome, complications, morbidity, quality of life

Word count 3557

For peer review only

For peer review only - http://bmjopen.bmj.com/site/about/guidelines.xhtml

Abstract

Background

Operative mortality for paediatric cardiac surgery has fallen to very low levels and currently stakeholders believe that this important safety outcome should be supplemented by a wider range of measures.

Objectives

To identify and define surgical morbidities considered by patients and clinicians to be most important to monitor routinely; to prospectively measure the incidence of morbidity over 21 months; and to evaluate the clinical and health-economic impact of morbidity over 6-months.

Design

The design was a prospective, multi-centre, multidisciplinary mixed methods study.

Setting

The setting was five of the ten paediatric cardiac surgery centres in the United Kingdom.

Participants

The participants were children under the age of 17 years with heart diseases.

Results

Families and clinicians prioritized these morbidities:

Acute neurological event, unplanned re-intervention, feeding problems, renal replacement therapy, major adverse events, extracorporeal life support, necrotising enterocolitis, post-surgical infection, and prolonged pleural effusion or chylothorax.

Amongst 3090 consecutive surgeries there were 675 (21.8%) with at least one morbidity. Independent risk factors for morbidity included neonatal age, complex heart disease and prolonged cardiopulmonary bypass time ($P<0{\cdot}001$). Amongst patients with morbidity, 6-month survival was 89·2% (95% CI 85·4, 90·6) compared to 99·1% (95% CI 98·6, 99·4) with no morbidity ($P<0{\cdot}001$).

Follow up for impact of morbidity amongst 666 patients over 6 months indicated that:

- Morbidity related impairment in health related quality of life at 6 weeks post procedure improved.

For peer review only

For peer review only - http://bmjopen.bmj.com/site/about/guidelines.xhtml

- There were a median of 13 (95% CI (10.2, 15.8) P<0·001) fewer days at home with morbidity.
- Morbidities were costly: ECLS carried an adjusted mean increase in hospital cost of £62,452 and multi-morbidity of £33,147 compared to no morbidity (P<0·01).

Conclusions

Evaluation of post-operative morbidity is inherently complex, but offers stakeholders considerably more information about paediatric cardiac surgery outcomes than the current metric of 30-day mortality.

Strengths and limitations of this study

- The study identifies early paediatric cardiac surgical morbidities that are considered to be important by both lay people and clinicians.
- The study prospectively measures the incidence of the defined morbidities in children undergoing heart surgery across five centres.
- Based on data collected, the study explores the risk factors for paediatric cardiac surgery morbidity statistically.
- Within a subset of patients, the impact of morbidities on patients and families are measured over 6 months in terms of quality of life, days spent at home rather than in hospital and hospital costs.

Study registration

The study has ethical approval from London City Road Research Ethics Committee (14-LO-1442).

Funding details

This project was funded by the National Institute for Health Research Health Services and Delivery Research programme (Project No: 12/5005/06). The views and opinions expressed therein are those of the authors and do not necessarily reflect those of the NIHR HS&DR programme or the Department of Health. The funder had no role in the study.

Conflicts of interest

None declared by authors.

For peer review only - http://bmjopen.bmj.com/site/about/guidelines.xhtml

Background

Over 5000 paediatric cardiac surgery procedures are performed in the UK each year and early survival has improved to over 98% since comprehensive national audit commenced in 2000. [1,2] Most stakeholders including clinicians, commissioners and users believe that whilst these early survival rates remain an important safety measure, it is has become imperative to explore a broader range of measures for outcome in this complex field of practice. Whilst there has been considerable research related to measuring, understanding and reducing peri-operative mortality for paediatric cardiac surgery, [3-5], much less attention has been focussed on surgical morbidities.

Morbidity is defined as a state of being unhealthy, or of experiencing an aspect of health that is ‘generally bad for you’. In this project, by ‘surgical morbidity’ we mean a defined aspect of ill health associated with a specific operation. In our study, we aimed to identify which surgical morbidities present the greatest burden on patients and health services following paediatric cardiac surgery. Views may differ between professionals and non-professionals as to what the term morbidity exactly refers to and which surgical morbidities are most important. [6] Therefore we set out to combine patient and carers’ perspectives with those of professional groups in defining a prioritised list of morbidities for prospective evaluation.

Over four years we:

1. Identified and defined nine surgical morbidities following paediatric heart surgery, taking into account views from patients, carers, psychologists, nurses and clinicians, which together captured important aspects of the clinical and health-economic burden;
2. Measured incidence of the defined morbidities in the UK patient population and in subgroups defined by case complexity;
3. Evaluated the impact of defined morbidities on quality of life and estimated their clinical and health economic burden.

For peer review only - http://bmjopen.bmj.com/site/about/guidelines.xhtml

Methods

Design and Setting

Our multidisciplinary mixed methods study took place across five UK paediatric cardiac surgery centres representing a range of programme sizes, which care for half of all patients nationally.

Participants

The participants were children under 17 years old with congenital heart disease (CHD).

1: Which morbidities?

Between January 2014 and September 2015 we reviewed existing literature, ran three family focus groups and undertook a family online discussion forum moderated by the Children's Heart Federation (a user group). Transcripts were thematically analysed and the resultant themes, together with the literature, helped to inform a long list of candidate surgical morbidities to consider. A multi-disciplinary group with patient and carer representation then ranked and selected a list of nine key morbidities using the nominal group technique and secret voting (6). This 'selection panel' [7] was informed in turn by clinical views on definitions and feasibility of routine monitoring for each candidate morbidity provided by an independent 'definition panel' [8] as reported in previous publications.

2. Incidence of morbidity

Measurement

Between October 2015 and June 2017, we measured morbidity incidence in consecutive surgical admissions across the study sites. Morbidities were attributed to the immediately preceding cardiac surgery and defined within the same hospitalisation, except for unplanned re operation which was defined as an unanticipated procedure within 30-days (see [8] for details) and mediastinitis which could be identified post-discharge by the operating surgeon (part of post-surgical infection morbidity see [8] for details). Data were regularly checked for completeness, clinical congruence and accuracy and, a three month sample of data was independently validated against the national audit data.

As for the UK audit of 30-day mortality, [9] all procedures more than 30 days apart on the same patient were included in the morbidity analysis. As a secondary outcome, we checked each patient's survival status at 6 months after first appearance in the dataset, based on National Congenital Heart Diseases Audit (NCHDA) [2] and local hospital records.

For peer review only - http://bmjopen.bmj.com/site/about/guidelines.xhtml

Analysis

We used multilevel logistic regression analysis to explore the role of pre-operative, patient-level case mix factors on the occurrence of any morbidity versus no morbidity, accounting for multiple procedures within patients. Clinical risk factor groups were derived from the finer diagnostic coding, based on previous peer reviewed research by our group. [9 10]

A univariate model to predict risk of any morbidity vs none was fitted for each risk factor. The estimated Odds ratios (OR) are presented along with 95% confidence intervals (CI). All factors significant on univariate analysis ($p<0 \cdot 1$) were considered in a multivariable model.

We used multiple imputation by chained equations to account for missing data. The imputation model included all risk factors considered in the univariate analysis, which we assumed included all predictors of missingness (missing data are indicated in our results). The multivariable models were derived by fitting a regression model for all significant predictors and estimates were combined using Rubin's rules. [11]

The secondary outcome of survival at 6 months was compared between morbidity groups using logistic regression. All analyses were performed in Stata 14. [12]

4. Impact of morbidity

Recruitment

For our study of impact, we attempted to recruit all patients with morbidity (as defined [8]) where at least one parent spoke reasonable English, and the family were resident in the UK. Where feasible, for each case we recruited a patient with no morbidity, matched on treating centre, age and univentricular status. Recruitment ran between October 2015 and June 2017 for all but one site where it stopped after 6 months due to resource constraints. The recruitment strategy led to a balanced sample in terms of morbidity patients and those with no morbidity (see results for detail), however we could not match every patient with a control. We included the available data on children who subsequently died.

Measures of impact

We evaluated the impact of morbidities over the 6 months immediately following surgery on the following outcomes:

a) Quality of life and psychological burden on children and parents

We used age-specific measures at 6 weeks and 6 months following surgery:

For peer review only - http://bmjopen.bmj.com/site/about/guidelines.xhtml

The PedsQL subscales (physical and psychosocial) and total scores, which range from 0 and 100, a higher score indicating better quality of life. PedsQL scores in a normal healthy population vary by age; expected scores for infants 0-12 months (which encompasses the median age for our cohort) are mean physical 84·98 (SD 9·45), mean psychosocial 80·47 (SD 12·64) and mean total 82·47 (SD 9·95). [13-15]

For their parents, we used the PHQ-4, which comprises four questions, measuring anxiety and depression in adults. Individual items are scored from zero to three. Scores ≥three for the first two questions suggest anxiety, and scores ≥three for last two questions suggest depression. [16] In the normal population, 4·8% have scores suggestive of anxiety and 6·6% have PHQ-4 scores suggestive of depression. [17]

We used mixed effects regression models for PedsQL/PHQ-4 results to compare the impact of morbidity (any versus none) on outcome. All models were adjusted for clustering within matched pairs and significant covariates associated with incidence of morbidity. The inclusion of hospital site made negligible difference to the results and is not included in our results.

We used multiple imputation, by chained equations to account for missing data for those patients known to be alive at 6 weeks and 6 months respectively.

b) Days at home over 6 months as an additional measure of disruption to family life

Length of inpatient stay following the index procedure was defined as the number of days between the operation and the date of discharge from the tertiary centre. Hospital stays that exceeded the 6-month follow-up period were censored after 183 days. All subsequent hospital admissions were captured and the total days spent in hospital including tertiary and secondary care admissions was subtracted from 183 days to generate a value for days at home by 6 months. Children who died in hospital were assigned a value of zero for this outcome.

We used quantile regression to estimate the effects of individual morbidities in terms of differences in medians since the measure was negatively skewed. Models were adjusted as described above. Given the high level of data completeness imputation was not used.

c) Costs of the index hospitalisation

We calculated the cost of the inpatient stay in the tertiary centre following the index surgical procedure as a key indicator of the economic burden to the hospital provider (measured in 2016/17 UK£).

For peer review only - http://bmjopen.bmj.com/site/about/guidelines.xhtml

Costs were calculated as the summation of days in intensive care unit (ICU), days on the ward, and all operating procedures within the defined period. For ICU stays, data were recorded on the level of care the patient received each day (Enhanced Care, High Dependency, High Dependency Advanced, ICU Basic, ICU Basic Enhanced, ICU Advanced, ICU Advanced Enhanced and ECLS). [18] Unit costs of ICU stays were daily costs for each level of care (ranging from £870 to £5440) applied to the number of days spent receiving that level of care. Unit costs per day for ward stays were obtained from the highest recruiting centre and varied according to the age of the patient (<two years, £904; ≥two years, £680). Unit costs for surgical procedures were costs per minute supplied by study sites; these varied by site and so we used the value for the site that recruited the largest number of patients to this study (£13·39 per minute), which was similar to the mean value across all sites.

We modelled the relationship between morbidities and cost of index hospitalisation, adjusted for significant covariates associated with hospital costs. Costs were skewed and to account for this we used a generalised linear model (GLM) with gamma family and log link, which has been recommended for modelling positively skewed data. [19] Missing outcome values were imputed using an iterative Markov chain Monte Carlo procedure based on multivariate normal regression, generating 20 imputed datasets.

Patient sample size

We anticipated that between 3000 and 3300 surgical patients would be admitted across the five sites during the study period. [2] We assumed that a clinically relevant difference in quality of life between matched pairs corresponded to a mean difference in quality of life score of at least 0.5 standard deviations. [20] To detect such a difference for PedsQL responses at 5% significance with 80% power requires a minimum of 32 matched pairs. Allowing for a 10% loss to follow-up rate, we aimed to recruit 36 matched pairs for each patient with morbidity, giving 80% power to detect a significant effect for any morbidity with a prevalence of at least 1·5%.

Patient and public involvement

The patient or user perspective lies at the core of the study methodology. Specifically, a key goal of the study was to consider the views of patients and parents when measures of morbidity are selected for future audit and benchmarking, in particular since emphasis may potentially differ between professionals and parents / patients.

For peer review only - http://bmjopen.bmj.com/site/about/guidelines.xhtml

Patient and family representatives from the Children's Heart Federation (CHF) were involved in aspects of the study design including design and running of the focus groups and the online discussion forum.

Representatives of the CHF participated in the facilitated nominal group meetings to select morbidities for inclusion in the incidence and impact studies.

A member of the CHF and a parent representative sat on the project steering group.

Results

1: Which morbidities?

Our final selected [7] and defined [8] list of included morbidities were:

Acute neurological event (ANE), unplanned re-intervention (URO), feeding problems (Feed), renal replacement therapy (Renal), major adverse events (MAE), extracorporeal life support (ECLS), necrotising enterocolitis (NEC), post-surgical infection (SSI) and prolonged pleural effusion or chylothorax (PPE).

We treated children with more than one of these events as a distinct group referred to as '*multi-morbidity*'. Recognising ECLS as a very severe event, which nearly always occurs with other morbidities, we treated ECLS as standalone morbidity (and always excluded from the multiple morbidity group). [21-25]

There was some divergence between the views of clinicians and families about the fundamental issue of what the important morbidities linked to paediatric cardiac surgery are. Health professionals from tertiary centres tended to prioritise clearly clinical issues related to the heart (e.g. use of ECLS and re operation) whereas parents placed greater emphasis on holistic outcomes for their child (e.g. feeding difficulties and child developmental problems). [7]

2. Incidence of morbidity

Descriptive results

We collected data on 3248 cardiac procedures and analysed 3090, after excluding 99 cardiac re-operations (a morbidity outcome) and 59 procedures that did not meet inclusion criteria.

The incidence of individual morbidities in isolation and overall for each is shown in Figure 1.

For peer review only - http://bmjopen.bmj.com/site/about/guidelines.xhtml

Figure 1 Incidence of morbidities

The figure shows the distinction between occurrence as a single morbidity and occurrence in combination with other morbidities, with 95% confidence intervals.

For peer review only

Significant risk factors for morbidity

As shown in Table 1, the most important risk factor for occurrence of ‘any morbidity’ was young age, particularly neonates, who were approximately five times as likely to experience a morbidity compared to children over one year old. Children with more complex heart disease or children whose operation included a bypass time longer than 90 minutes were almost twice as likely to experience morbidity. Other important independent risk factors were: palliative or staged procedures, the presence of functionally univentricular heart and pre-procedure severity of illness factors (e.g. pre-procedure mechanical ventilation or shock).

For peer review only

Table 2 Summary of risk factors by any morbidity outcome

The weight was missing in 87 (2·8%) procedures and weight for age was set to missing for an additional 99 (3·2%) patients in whom the value was >5 SD from the normative mean. Other data were complete. Values include imputed data.

Cardiac diagnosis group A) hypoplastic left heart syndrome, Truncus arteriosus, pulmonary atresia intact septum, B) Functionally univentricular heart, pulmonary atresia ventricular septal defect, C) transposition of the great arteries all types, interrupted aortic arch, totally anomalous pulmonary venous connection, D) patent ductus arteriosus, tricuspid valve anomalies, acquired heart disease, complete atrioventricular septal defect, E) Tetralogy of Fallot, mitral valve anomalies, isolated aortic stenosis, aortic regurgitation, aortic arch obstruction, subaortic obstruction, ventricular septal defect, atrial septal defect.

	No morbidity (N = 2415)	Any Morbidity (N=675)	Univariate odds ratio (95% CI), P Value	Multivariable odds ratio(95% CI), P Value
**Male**	1299 (53.8)	372 (55.1)	1.05 (0.89, 1.25), P=0.54	-
**Median Age (days) (IQR)**	286 (105, 1582)	102 (10, 331)		
**Child**	1111 (46.0)	160 (23.7)		
**Infant**	1023 (42.4)	268 (39.7)	1.82 (1.47, 2.25), P<0.001	1.61 (1.26, 2.05), P<0.001
**Neonate**	281 (11.6)	247 (36.6)	6.10 (4.81, 7.75)< P<0.001	5.26 (3.90, 7.09), P<0.001
**Median Weight (Kg) (IQR)**	7.7 (4.7, 16.2)	4.6 (3.2, 8.0)		
**Low weight <2 SD below mean**	714 (31.5)	234 (36.8)	1.27 (1.05, 1.52)· P=0.01	1.21 (0.97, 1.51), P=0.09
**Cardiac Diagnosis Group**				
**E Least complex (reference)**	1002 (41.5)	123 (18.2)		
**D**	796 (33.0)	227 (33.6)	5.13 (3.79, 6.93), P<0.001	2.02 (1.58, 2.60), P<0.001
**C**	215 (8.9)	109 (16.2)	3.83 (2.85, 5.13), P<0.001	1.44 (1.00, 2.07), P=0.05
**B**	232 (9.6)	109 (16.2)	4.13 (3.07, 5.55), P<0.001	2.62 (1.85, 3.71), P<0.001
**A Most Complex**	170 (7.0)	107 (15.8)	2.32 (1.83, 2.94), P<0.001	2.14 (1.41, 3.24), P<0.001
**Functionally univentricular heart**	255 (10.6)	159 (23.6)	2.61 (2.11, 3.23), P<0.001	1.55 (1.07, 2.24), P=0.02
**Acquired comorbidity**	337 (14.0)	119 (17.6)	1.32 (1.05, 1.66), P=0.02	1.33 (1.03, 1.71), P=0.03
**Congenital comorbidity**	537 (22.2)	178 (26.4)	1.25 (1.03, 1.52), P=0.03	1.28 (1.02, 1.59), P=0.03
**Severity of illness indicators**	222 (9.2)	152 (22.5)	2.87 (2.30, 3.58), P<0.001	1.52 (1.16, 2.00), P<0.01
**Premature <37 weeks gestation**	231 (9.6)	73 (10.8)	1.15 (0.87, 1.51), P=0.33	-
**Downs Syndrome**	214 (8.9)	63 (9.3)	1.06 (0.79, 1.43), P=0.71	-
**Additional cardiac risk**	165 (6.8)	65 (9.6)	1.45 (1.09, 1.94), P=0.01	1.39 (0.99, 1.94), P=0.05
**Surgical procedure type**				
**Reparative/corrective (reference)**	1391 (57.6)	332 (49.2)		
**Palliative/staged**	331 (13.7)	179 (26.5)	2.27 (1.82, 2.82), P<0.001	1.65 (1.14, 2.38), P<0.01
**Ungrouped**	693 (28.7)	164 (24.3)	0.99 (0.81, 1.22), P=0.94	1.04 (0.82, 1.31), P=0.75
**Median bypass time (mins) (IQR)**	72 (42, 110)	110 (62, 156)		
**No bypass (reference)**	390 (16.2)	103 (15.3)		
**Up to 90 mins**	1148 (47.5)	150 (22.2)	0.48 (0.35, 0.65), P<0.001	0.78 (0.57, 1.09), P=0.14
**More than 90 mins**	877 (36.3)	422 (62.5)	1.76 (1.32, 2.34), P<0.001	2.28 (1.67, 3.12), P<0.001

For peer review only - http://bmjopen.bmj.com/site/about/guidelines.xhtml

Secondary outcomes

Patient survival was highest and patient hospital stay was shortest for those with no morbidity (see Table 2). The 6-month survival was significantly lower for patients with a single morbidity, for those with 'ECLS' and for 'multi-morbidity' patients, compared with patients with no morbidity P < 0·001 for all.

Table 2 Secondary outcomes of hospital stay and survival at 6 months by morbidity

The number (%) of morbidities was based on individual surgical episode, as was the length of stay, whereas survival was calculated per patient. Data are presented based on morbidity in isolation, multi-morbidity and ECLS. Length of stay data was missing for 9 patients and Life status was missing in 16 patients who were not included.

Morbidity type	Number (%)	Median length of post-operative hospital stay in days (IQR)	6 month survival Number, %, 95% CI
**No morbidity**	2415 (78.2%)	8 days (IQR 5, 13)	2202/2217 99.3% (98.9, 99.6)
**Acute neurological event**	14 (0.5)	19 days (12, 39)	12/13 92.3% (64.0, 99.8)
**Unplanned re-intervention**	59 (1.9%)	22 days (14, 33)	50/54 92.6% (82.1, 97.6)
**Feeding problems**	99 (3.2%)	20.5 days (12, 36)	90/91 98.9% (94, 100)
**Renal support**	40 (1.3%)	17 days (14, 26)	37/39 94.9% (82.7, 99.4)
**Major adverse event**	34 (1.1)	16.5 days (8, 25)	28/33 84.9% (68.1, 94.9)
**Necrotising enterocolitis**	32 (1.0%)	24.5 days (18.5, 49.5)	28/30 93.3% (77.9, 99.2)
**Post-surgical infection**	27 (0.9%)	20.5 days (11, 28)	25/25 100% (86.3, 100)
**Prolonged pleural effusion**	111 (3.6%)	20 days (14, 28)	95/96 99.0% (94.3, 100)
**Multi- morbidity**	197 (6.4)	35 days (IQR 22, 56)	158/190 83.2% (77.1, 88.2)
**Extracorporeal life support**	62 (2/0%)	43 days (IQR 20, 84)	31/57 54.4% (40.7, 67.6)

3. Impact of morbidity

Patient sample

We recruited 60% of eligible families, amounting to 666 children of whom 340 (51%) had at least one morbidity. Of these, 19 died within 30 days and 39 died within 6 months. We pair matched 558 patients and 108 patients unmatched of whom 61 had morbidity; all of the patients were included in the statistical analysis.

Quality of life and anxiety - depression outcomes

For the 6-weeks data a comparison between patients with missing versus non missing data indicated the only difference was in the proportions with severity of illness factors and additional cardiac risk factors. Importantly there was no difference in the proportion of patients with or without morbidity. At the 6 months mark there was no difference in any baseline factor or morbidity occurrence.

For peer review only - http://bmjopen.bmj.com/site/about/guidelines.xhtml

All PedQL scores were lower than in a healthy population (see methods [13-15] and Table 3). In terms of our study objective to assess the impact of morbidity; the total PedsQL scores were significantly reduced for children with any morbidity at 6 weeks in comparison to children with no morbidity (case mix adjusted score reduction of -5·2 (95% CI -8·3, -2·2) P<0·01). On a positive note, this difference narrowed to small non-significant reduction in total score by 6 months. Physical scores were affected more than psychosocial scores, and were still significantly impaired at 6-months.

All parents experienced higher rates of both anxiety and depression than a healthy population (see methods and Table 3). The parents of children with morbidity were around 57% more likely to experience anxiety; and around 77% more likely to experience depression, at 6 weeks post operation than parents of children without morbidity. PHQ-4 scores had improved by 6 months, and although there remained a higher chance of both anxiety and depression with morbidity, the difference narrowed and was not significant.

Table 3 Quality of life impact

Quality of life outcome was based on PedsQL data for 478 children at 6-weeks (26% missing) and 403 children at 6 months (36% missing). Parental anxiety and depression was assessed based on the PHQ-4 responses of 481 parents at 6 weeks (26% missing) and 394 parents at 6 months (37% missing). Values include imputed data.

**Time point**	**6 weeks**	**6 weeks**	**6 weeks**
**Group and parameter**	*No Morbidity* (n=242) *Score Mean (SD)*	*Any Morbidity* (n=236) *Score Mean (SD)*	*Adjusted difference in mean score (95% CI), P value*
**Peds QL Physical**	79.0 (16.2)	69.1 (21.8)	-8.3 (-11.8, -4.9), P<0.001
**Peds QL Psychosocial**	79.4 (14.7)	75.2 (19.0)	-2.7 (-5.9, 0.5), P=0.08
**Peds QL Total**	79.3 (13.8)	72.1 (19.1)	-5.2 (-8.3, -2.2), P<0.01
**Time point**	**6 months**	**6 months**	**6 months**
**Group and parameter**	*No Morbidity (n=208) Score Mean (SD)*	*Any Morbidity (n=195) Score Mean (SD)*	*Adjusted difference in mean score* (95% CI)
**Peds QL Physical**	82.3 (16.6)	76.6 (18.6)	-4.2 (-7.6, -0.8), P=0.02
**Peds QL Psychosocial**	78.0 (14.7)	76.4 (15.4)	-0.9 (-3.5, 1.8), P=0.5
**Peds QL Total**	79.8 (13.9)	76.6 (15.0)	-2.3 (-4.9, 0.3), P=0.08
**Time point**	**6 weeks**	**6 weeks**	**6 weeks**
**Group and parameter**	*No Morbidity (n=232) Number (%) with attribute*	*Any Morbidity (n=250) Number (%) with attribute*	*Adjusted odds ratio for anxiety/ depression* (95% CI)
**PHQ-4 Anxiety**	55 (23.7)	92 (36.8)	1.57 (1.04, 2.35), P=0.03
**PHQ-4 Depression**	26 (11.2)	55 (22.1)	1.77 (1.10, 2.86), P=0.02
**Time point**	**6 months**	**6 months**	**6 months**
**Group and parameter**	*No Morbidity (n=200) Number (%) with attribute*	*Any Morbidity (n=195) Number (%) with attribute*	*Adjusted odds ratio for anxiety/ depression (95% CI)*
**PHQ-4 Anxiety**	23 (11.5)	34 (17.4)	1.37 (0.77, 2.43), P=0.28
**PHQ-4 Depression**	15 (7.5)	23 (11.9)	1.45 (0.79, 2.68), P=0.23

For peer review only - http://bmjopen.bmj.com/site/about/guidelines.xhtml

Days at home by 6 months outcome

Children with no morbidity had a median of 174 days at home (IQR 169, 176) out of a possible 183 days in 6 months. The presence of any morbidity reduced the median days at home by 13 days over 6 months (95% CI (10·2, 15·8) P<0·001). When individual morbidities were each considered in turn, the difference in the case mix adjusted median days at home was significantly lower at 6 months for all of them apart from 'renal support' (P<0.05).

Health economic outcome

Costs of the index hospitalisation were available for 613 patients (8% missing). These indicated a mean value of £36,038 (SD £42,588), and a median value of £21,696 (IQR £14,309 to £36,161).

In Table 4, the regression results are presented in terms of marginal effects, which represent the adjusted incremental cost linked to the stated morbidity in comparison to the 'no morbidity' category for whom, there was a mean cost per patient £21,292. The greatest adjusted cost difference to those with no morbidity was for 'ECLS', where the adjusted mean difference in costs was £62,452; followed by 'multi-morbidity' where the adjusted mean difference was £33,147 (P<0·01 for both). Significantly increased (P>0·05) costs were also found for 'unplanned reoperation', 'feeding problems', 'renal support' and 'prolonged pleural effusion'. The remaining morbidities, representing the lowest number of patients per category, showed non-significant differences.

Table 4 Cost of hospital stay following index surgical procedure by morbidity

Values include imputed data. Figures in the Marginal Effect column are the mean difference in costs between each morbidity category and the 'no morbidities' category (mean cost per patient £21,292) conditional on the covariates. Costs are in 2016/17 UK£.

Morbidity	Coef.	Marginal Effect (£) (95% CI), P Value
**Acute neurological event (n=6)**	0.50	13,911 (-3,923, 50,059), P=0.16
**Unplanned re-intervention (n=26)**	0.62	18,330 (7,475, 33,282), P<0.01
**Feeding morbidity (n=45)**	0.55	15,541 (7,200, 26,326), P<0.01
**Renal support (n=24)**	0.41	10,798 (1,846, 23,215), P=0.01
**Major adverse event (n=22)**	0.30	7,555 (-1,131, 19,984), P=0.10
**Necrotising enterocolitis (n=11)**	0.37	9,547 (-2,173, 28,454), P=0.13
**Post-surgical infection (n=11)**	0.27	6,466 (-3,947, 23,130), P=0.17
**Prolonged pleural effusion (n=50)**	0.33	8,177 (1,444, 16,904), P=0.01
**Extracorporeal life support (n=27)**	1.37	62,452 (39,546, 93,983), P<0.01

For peer review only

**Multi-morbidity (n=118)**	0.94	33,147 (23,669, 44,624)· P<0.01

Discussion

Overview of findings

This paper presents the results of the first prospective multi-centre study of paediatric cardiac surgery morbidity over 6-months after operation. The results are novel and indicate that evaluation of morbidity adds considerably to the current lone metric of 30-day survival.

We note the excellent outcomes in children who had no morbidities: their survival at 6-months post operation was 99·3% and their median length of stay was 8 days, suggesting that the morbidities we selected do capture most of the complication-related adverse outcomes for this context.

Based on our impact sub study, 'ECLS' and 'multiple morbidities' are particularly important adverse outcomes – they are associated with high mortality, high resource use and lower quality of life for surviving patients. Future routine monitoring and public reporting for these should be considered by centres and the national audit.

In our engagement work, patients' families consistently told us that outcomes affecting the child as a whole over the medium or longer term are very important to them. We thus included morbidities such as feeding problems and acute neurological events but note that those types of outcome were the most complicated to capture, and we intend to publish additional material on these topics.

Limitations

Definition, measurement, reporting and interpretation of morbidity, is much more complex than for mortality and requires significant staff resources and clinician buy in. Some morbidities are more challenging to measure and to interpret (see specific morbidities). The number of individual morbidities was low in some of our analyses limiting interpretation.

Context

There have been previous attempts at reporting of paediatric cardiac surgery morbidity outcomes: two single centre studies generated an aggregate 'Morbidity Index' by assigning subjective weights to post-operative complications [26 27] and the Society of Thoracic Surgery in the USA developed a 'Morbidity Score' based on data from their multi-centre registry. [28] Condensing diverse morbidities into a single score loses information and recent work on

For peer review only - http://bmjopen.bmj.com/site/about/guidelines.xhtml

using graphical methods to routinely monitor a range of morbidities [29] highlighted the complexity of graphically summarising multiple morbidities (see also commentary by Utley, Brown and Tsang [30]). The Pediatric Cardiac Critical Care Consortium (PC^4) was set up in 2009, aiming to improve the quality of care for patients with critical paediatric and congenital cardiovascular disease in North America and abroad. The PC^4 database provides partner sites with access to real-time, reliable and actionable data to be used for local quality improvements and has been the basis for a range of excellent publications related to complications after paediatric cardiac surgery. [31-34] However membership of PC^4 is voluntary for institutions on a subscription basis, the reported measures whilst of great importance have all been selected by clinicians and the reporting of outcomes is accessible only to subscribing member institutions.

Future practice and research

Specific morbidities

Neurodevelopmental problems are common in children with CHD undergoing surgery [35] however we detected acute neurological events (ANE) in only 2·1% of patients: it appears that peri-operative 'ANE' represents the tip of the iceberg and hence the scope of surveillance for child neurodevelopment in CHD needs to extend well beyond the peri-operative period.

Despite their great important to families, we note that participants reported subjectivity in the collection of data on 'feeding problems'; hence these were difficult to capture consistently. Additional research may help determine the best way to alleviate the impact of feeding problems in CHD.

Our measure of renal failure was the need for renal support, and although relatively easy data to collect this may not be the optimal method to capture this morbidity, given that there are differences in practice between clinicians. [36] Further research may help us to understand the best approach to manage post-operative renal injury in CHD.

Feeding back to stakeholders

We used study data collected during the course of the morbidity project, to co-develop parent and carer information resources, showing what the morbidities are and how their incidence and the length of stay may vary based on the complexity of the child's condition. Parents told us that it helps to know that; *firstly* they are not alone in facing a complication, *secondly* clinical teams have seen complications before and know how to deal with them, and *thirdly* that it is better as a parent to 'be prepared'. Furthermore, parents indicated that information

For peer review only - http://bmjopen.bmj.com/site/about/guidelines.xhtml

about impact such as *nearly all children who experience a complication and recover will have a similar quality of life to children who did not experience a complication by the six month mark,* was very helpful to know.

Within the scope of this project, a new Excel tool has been developed and piloted, which enables clinical teams to benchmark and report the local rates of morbidities with a quality assurance goal, such as in a mortality and morbidity conference. Furthermore, we have kept in close contact with the National Congenital Heart Diseases Audit (NCHDA) and the Clinical Reference Group for CHD services. NCHDA has already started to collect five of the nine morbidities within the nationally mandated dataset, using the definitions that we developed.

Author contributions

Designed the study and wrote the protocol: Katherine Brown, Christina Pagel, Martin Utley, Deborah Ridout, Jo Wray, Steve Morris, and Victor Tsang.

Clinical expertise: Victor Tsang, David Anderson, David Barron, Serban Stoica, Andrew Maclean, Katherine Brown, Shane Tibby, David Barron, Peter Davis and Warren Rodrigues.

Study implementation, protocol adjustments, data cleaning: Katherine Brown, Alison Jones, Karen Sheehan, Victor Tsang, Jane Cassidy, David Barron, Peter Davis, Serban Stoica, Warren Rodrigues, and Shane Tibby.

Statistical analysis: Deborah Ridout, Christina Pagel, Shane Tibby, and Katherine Brown.

Health economic analysis: Emma Hudson, Steve Morris.

Contributed to the write up and signed off the paper: Katherine Brown, Christina Pagel, Deborah Ridout, Jo Wray, Victor Tsang, David Anderson, David Barron, Jane Cassidy, Peter Davis, Emma Hudson, Alison Jones, Andrew McLean, Steve Morris, Warren Rodrigues, Karen Sheehan, Serban Stoica, Shane Tibby, and Thomas Witter.

Acknowledgements (includes patient and public contributor names)

We would like to thank Martin Utley, Sheryl Snowball, Luke Maidment, Sarah Bohannon, Liz Smith, Kate Penny-Thomas, Joanne Webb, Sinead Cummins, John Stickley, Natasha Khan, Teresa Dickson, Ray Samson, Isobel Mcleod, Paul Wellman, Rhian Lakhani, Kathleen Selway, Carrie Cherrington, Andrew Parry, Rob Tulloh, Bill Gaynor, Rodney Franklin, Lisa

For peer review only - http://bmjopen.bmj.com/site/about/guidelines.xhtml

Allera, Kate Bull, Trevor Ritchens, Branko Mimic, Jon Smith, Lyvonne Tume, Vibeke Hjortdal, Michael Vath, Tom Treasure, Anne Keatley Clarke, Bea Tuten, and all the patients who participated in the study.

For peer review only

For peer review only - http://bmjopen.bmj.com/site/about/guidelines.xhtml

References

1. Brown KL, Crowe S, Franklin R, et al. Trends in 30-day mortality rate and case mix for paediatric cardiac surgery in the UK between 2000 and 2010. Open Heart 2015;**2**(1):e000157.
2. NICOR. NICOR: National Institute for Cardiovascular Outcomes Research: Congenital Heart Diseases Website. Secondary NICOR: National Institute for Cardiovascular Outcomes Research: Congenital Heart Diseases Website 2017. www.nicor4.nicor.org.uk/CHD.
3. O'Brien SM, Clarke DR, Jacobs JP, et al. An empirically based tool for analyzing mortality associated with congenital heart surgery. J Thorac Cardiovasc Surg 2009;**138**(5):1139-53.
4. Jenkins KJ, Gauvreau K, Newburger JW, et al. Consensus-based method for risk adjustment for surgery for congenital heart disease. J Thorac Cardiovasc Surg 2002;**123**(1):110-8.
5. Lacour-Gayet F, Clarke D, Jacobs J, et al. The Aristotle score for congenital heart surgery. Semin Thorac Cardiovasc Surg Pediatr Card Surg Annu 2004;**7**:185-91.
6. Cooke M, Thackray S. Differences between community professional and patient perceptions of chronic obstructive pulmonary disease treatment outcomes: a qualitative study. J Clin Nurs 2012;**21**(11-12):1524-33.
7. Pagel C, Brown KL, McLeod I, et al. Selection by a panel of clinicians and family representatives of important early morbidities associated with paediatric cardiac surgery suitable for routine monitoring using the nominal group technique and a robust voting process. BMJ open 2017;**7**(5):e014743.
8. Brown KL, Pagel C, Brimmell R, et al. Definition of important early morbidities related to paediatric cardiac surgery. Cardiol Young 2017;**27**(4):747-56.
9. Rogers L, Brown KL, Franklin RC, et al. Improving Risk Adjustment for Mortality After Pediatric Cardiac Surgery: The UK PRAiS2 Model. Ann Thorac Surg 2017;**104**(1):211-19.
10. Brown KL, Rogers L, Barron DJ, et al. Incorporating Comorbidity Within Risk Adjustment for UK Pediatric Cardiac Surgery. Ann Thorac Surg 2017;**104**(1):220-26.
11. Rubin DB. Multiple Imputation for Nonresponse in Surveys. In: Wiley, ed. New York, 1987.
12. 2015. S. Stata Statistical Software: Release 14. . In: LP. S, ed. College Station, TX:, 2015.
13. Varni JW, Seid M, Rode CA. The PedsQL: measurement model for the pediatric quality of life inventory. Med Care 1999;**37**(2):126-39.
14. Varni JW, Seid M, Kurtin PS. PedsQL 4.0: reliability and validity of the Pediatric Quality of Life Inventory version 4.0 generic core scales in healthy and patient populations. Med Care 2001;**39**(8):800-12.
15. Varni JW, Limbers CA, Neighbors K, et al. The PedsQL Infant Scales: feasibility, internal consistency reliability, and validity in healthy and ill infants. Qual Life Res 2011;**20**(1):45-55.
16. Kroenke K, Spitzer RL, Williams JB, et al. An ultra-brief screening scale for anxiety and depression: the PHQ-4. Psychosomatics 2009;**50**(6):613-21.
17. Lowe B, Wahl I, Rose M, et al. A 4-item measure of depression and anxiety: validation and standardization of the Patient Health Questionnaire-4 (PHQ-4) in the general population. Journal of affective disorders 2010;**122**(1-2):86-95.
18. https://www.picanet.org.uk/wp-content/uploads/sites/25/2018/05/PICANet_National_Report_2007.pdf. PCCMDS. Secondary PCCMDS 2018.
19. Barber J, Thompson S. Multiple regression of cost data: use of generalised linear models. Journal of health services research & policy 2004;**9**(4):197-204.
20. Norman GR, Sloan JA, Wyrwich KW. Interpretation of changes in health-related quality of life: the remarkable universality of half a standard deviation. Med Care 2003;**41**(5):582-92.

For peer review only - http://bmjopen.bmj.com/site/about/guidelines.xhtml

21. Aharon AS, Drinkwater DC, Jr., Churchwell KB, et al. Extracorporeal membrane oxygenation in children after repair of congenital cardiac lesions. Annals of Thoracic Surgery 2001;**72**(6):2095-101; discussion 101-2.
22. Alsoufi B, Shen I, Karamlou T, et al. Extracorporeal life support in neonates, infants, and children after repair of congenital heart disease: modern era results in a single institution. Annals of Thoracic Surgery 2005;**80**(1):15-21; discussion 21.
23. Beiras-Fernandez A, Deutsch M-A, Kainzinger S, et al. Extracorporeal membrane oxygenation in 108 patients with low cardiac output - a single-center experience. International Journal of Artificial Organs 2011;**34**(4):365-73.
24. Chauhan S, Malik M, Malik V, et al. Extra corporeal membrane oxygenation after pediatric cardiac surgery: a 10 year experience. Annals of Cardiac Anaesthesia 2011;**14**(1):19-24.
25. Coskun KO, Coskun ST, Popov AF, et al. Extracorporeal life support in pediatric cardiac dysfunction. Journal Of Cardiothoracic Surgery 2010;**5**:112.
26. Bojan M, Gerelli S, Gioanni S, et al. Evaluation of a new tool for morbidity assessment in congenital cardiac surgery. Ann Thorac Surg 2011;**92**(6):2200-4.
27. Costello JM, Cooper DS, Jacobs JP, et al. Intermediate-term outcomes after paediatric cardiac extracorporeal membrane oxygenation--what is known (and unknown). Cardiol Young 2011;**21 Suppl 2**:118-23.
28. Jacobs ML, O'Brien SM, Jacobs JP, et al. An empirically based tool for analyzing morbidity associated with operations for congenital heart disease. J Thorac Cardiovasc Surg 2012.
29. Salvin JW, Scheurer MA, Laussen PC, et al. Blood transfusion after pediatric cardiac surgery is associated with prolonged hospital stay. Ann Thorac Surg 2011;**91**(1):204-10.
30. Utley M, Brown K, Tsang V. Invited commentary. Ann Thorac Surg 2012;**94**(5):1602-3.
31. Alten JA, Rahman AF, Zaccagni HJ, et al. The Epidemiology of Health-Care Associated Infections in Pediatric Cardiac Intensive Care Units. Pediatr Infect Dis J 2017.
32. Hickey PA, Connor JA, Cherian KM, et al. International quality improvement initiatives. Cardiol Young 2017;**27**(S6):S61-S68.
33. Gaies M, Werho DK, Zhang W, et al. Duration of Postoperative Mechanical Ventilation as a Quality Metric for Pediatric Cardiac Surgical Programs. Ann Thorac Surg 2018;**105**(2):615-21.
34. Buckley JR, Graham EM, Gaies M, et al. Clinical epidemiology and centre variation in chylothorax rates after cardiac surgery in children: a report from the Pediatric Cardiac Critical Care Consortium. Cardiol Young 2017:1-8.
35. Marino BS, Lipkin PH, Newburger JW, et al. Neurodevelopmental outcomes in children with congenital heart disease: evaluation and management: a scientific statement from the American Heart Association. Circulation 2012;**126**(9):1143-72.
36. Kwiatkowski DM, Goldstein SL, Cooper DS, et al. Peritoneal Dialysis vs Furosemide for Prevention of Fluid Overload in Infants After Cardiac Surgery: A Randomized Clinical Trial. JAMA pediatrics 2017;**171**(4):357-64.

For peer review only - http://bmjopen.bmj.com/site/about/guidelines.xhtml

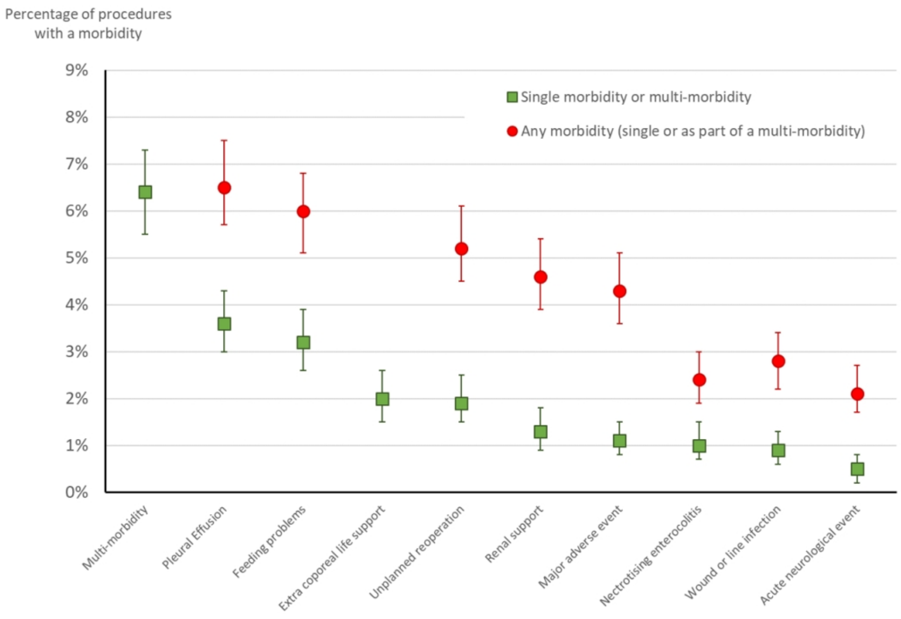

Figure 1 Incidence of morbidities
The figure shows the distinction between occurrence as a single morbidity and occurrence in combination with other morbidities, with 95% confidence intervals.

STROBE Statement—checklist of items that should be included in reports of observational studies

	Item No	Recommendation
**Title and abstract**	1	(*a*) Indicate the study's design with a commonly used term in the title or the abstract
		(*b*) Provide in the abstract an informative and balanced summary of what was done and what was found
**Introduction**		
Background/rationale	2	Explain the scientific background and rationale for the investigation being reported
Objectives	3	State specific objectives, including any prespecified hypotheses
**Methods**		
Study design	4	Present key elements of study design early in the paper
Setting	5	Describe the setting, locations, and relevant dates, including periods of recruitment, exposure, follow-up, and data collection
Participants	6	(*a*) *Cohort study*—Give the eligibility criteria, and the sources and methods of selection of participants. Describe methods of follow-up<br>*Case-control study*—Give the eligibility criteria, and the sources and methods of case ascertainment and control selection. Give the rationale for the choice of cases and controls<br>*Cross-sectional study*—Give the eligibility criteria, and the sources and methods of selection of participants
		(*b*) *Cohort study*—For matched studies, give matching criteria and number of exposed and unexposed<br>*Case-control study*—For matched studies, give matching criteria and the number of controls per case
Variables	7	Clearly define all outcomes, exposures, predictors, potential confounders, and effect modifiers. Give diagnostic criteria, if applicable
Data sources/ measurement	8*	For each variable of interest, give sources of data and details of methods of assessment (measurement). Describe comparability of assessment methods if there is more than one group
Bias	9	Describe any efforts to address potential sources of bias
Study size	10	Explain how the study size was arrived at
Quantitative variables	11	Explain how quantitative variables were handled in the analyses. If applicable, describe which groupings were chosen and why
Statistical methods	12	(*a*) Describe all statistical methods, including those used to control for confounding
		(*b*) Describe any methods used to examine subgroups and interactions
		(*c*) Explain how missing data were addressed
		(*d*) *Cohort study*—If applicable, explain how loss to follow-up was addressed<br>*Case-control study*—If applicable, explain how matching of cases and controls was addressed<br>*Cross-sectional study*—If applicable, describe analytical methods taking account of sampling strategy
		(*e*) Describe any sensitivity analyses

Continued on next page

For peer review only - http://bmjopen.bmj.com/site/about/guidelines.xhtml

**Results**		
Participants	13*	(a) Report numbers of individuals at each stage of study—eg numbers potentially eligible, examined for eligibility, confirmed eligible, included in the study, completing follow-up, and analysed
		(b) Give reasons for non-participation at each stage
		(c) Consider use of a flow diagram
Descriptive data	14*	(a) Give characteristics of study participants (eg demographic, clinical, social) and information on exposures and potential confounders
		(b) Indicate number of participants with missing data for each variable of interest
		(c) *Cohort study*—Summarise follow-up time (eg, average and total amount)
Outcome data	15*	*Cohort study*—Report numbers of outcome events or summary measures over time
		Case-control study—Report numbers in each exposure category, or summary measures of exposure
		Cross-sectional study—Report numbers of outcome events or summary measures
Main results	16	(*a*) Give unadjusted estimates and, if applicable, confounder-adjusted estimates and their precision (eg, 95% confidence interval). Make clear which confounders were adjusted for and why they were included
		(*b*) Report category boundaries when continuous variables were categorized
		(*c*) If relevant, consider translating estimates of relative risk into absolute risk for a meaningful time period
Other analyses	17	Report other analyses done—eg analyses of subgroups and interactions, and sensitivity analyses
**Discussion**		
Key results	18	Summarise key results with reference to study objectives
Limitations	19	Discuss limitations of the study, taking into account sources of potential bias or imprecision. Discuss both direction and magnitude of any potential bias
Interpretation	20	Give a cautious overall interpretation of results considering objectives, limitations, multiplicity of analyses, results from similar studies, and other relevant evidence
Generalisability	21	Discuss the generalisability (external validity) of the study results
**Other information**		
Funding	22	Give the source of funding and the role of the funders for the present study and, if applicable, for the original study on which the present article is based

*Give information separately for cases and controls in case-control studies and, if applicable, for exposed and unexposed groups in cohort and cross-sectional studies.

Note: An Explanation and Elaboration article discusses each checklist item and gives methodological background and published examples of transparent reporting. The STROBE checklist is best used in conjunction with this article (freely available on the Web sites of PLoS Medicine at http://www.plosmedicine.org/, Annals of Internal Medicine at http://www.annals.org/, and Epidemiology at http://www.epidem.com/). Information on the STROBE Initiative is available at www.strobe-statement.org.

For peer review only

BMJ Open

What are the important morbidities associated with paediatric cardiac surgery? : A mixed methods study

Journal:	*BMJ Open*
Manuscript ID	bmjopen-2018-028533.R1
Article Type:	Research
Date Submitted by the Author:	21-May-2019
Complete List of Authors:	Brown, Katherine; Great Ormond Street Hospital for Children, Cardiorespiratory Division<br>Pagel, Christina; UCL<br>Ridout, Deborah; UCL, Institute of Child Health<br>Wray, Jo; Great Ormond Street Hospital for Children, Cardiorespiratory Division<br>Anderson, David; Evelina London<br>Barron, David; Birmingham Women's and Children's NHS Foundation Trust<br>Cassidy, Jane; Birmingham Women's and Children's NHS Foundation Trust<br>Davis, Peter; Bristol Royal Hospital for ChilDr.en, Paediatric Intensive Care<br>Hudson, Emma; Health Economics, University College London<br>Jones, Alison; Birmingham Women's and Children's NHS Foundation Trust<br>Mclean, Andrew; Royal Hospital for Children, Congenital Heart Surgery<br>Morris, Stephen; University College London, Department of Applied Health Research<br>Rodrigues, Warren; Royal Hospital for Children Glasgow<br>Sheehan, Karen; Bristol Royal Hospital for Children<br>Stoica, Serban ; University Hospitals Bristol NHS Foundation Trust<br>Tibby, Shane; Evelina Childrens Hospital, Paediatric Intensive Care<br>Witter, Thomas; Evelina London Children's Healthcare<br>Tsang, Victor; Great Ormond Street Hospital for Children, Cardiorespiratory Division
**Primary Subject Heading**:	Surgery
Secondary Subject Heading:	Cardiovascular medicine
Keywords:	PAEDIATRICS, Cardiac surgery < SURGERY, complications, quality of life

SCHOLARONE™
Manuscripts

What are the important morbidities associated with paediatric cardiac surgery? : A mixed methods study

Authors

Katherine L Brown MD[1], Christina Pagel PHD[2], Deborah Ridout MSc[3], Jo Wray PHD[1], David Anderson FRCS[4], David J Barron MD[5], Jane Cassidy MRCP[5], Peter Davis FRCPCH[6], Emma Hudson MSc[8], Alison Jones MSc[5], Andrew McLean FRCS[10], Stephen Morris PHD[8], Warren Rodrigues MRCPCH[10], Karen Sheehan MSc[6], Serban Stoica FRCS[6], Shane Tibby MRCP[4], Thomas Witter RN[4], Victor T Tsang FRCS[1]

[1]Charles West Division, Great Ormond Street Hospital NHS Foundation Trust, Great Ormond Street, London

[2]Clinical Operational Research Unit, University College London, London

[3]Population, Policy and Practice Programme, UCL Great Ormond Street Institute of Child Health, London

[4]Department Paediatric Cardiology and Cardiac Surgery, Evelina London Children's Hospital, London

[5]Department of Intensive care and Paediatric Cardiac Surgery, Birmingham Children's Hospital, Birmingham

[6]Paediatric Intensive Care Unit and Department Paediatric Cardiac Surgery, Bristol Royal Hospital for Children, Bristol

[7]Paediatric Cardiology Department, Royal Brompton and Harefield NHS Foundation Trust, London

[8]Health Economics, Department of Applied Health Research, UCL

[9]Community Child Health, UCL Great Ormond Street Institute of Child Health, London

[10]Department of Intensive care, Royal Hospital for Children, Glasgow

Corresponding author

Katherine L Brown Great Ormond Street Hospital NHS Foundation Trust, Great Ormond Street, London WC1N 3JH

Katherine.brown@gosh.nhs.uk

Phone 0207 405 9200 extension 8016 or 8180

Fax 0207 829 8673

For peer review only - http://bmjopen.bmj.com/site/about/guidelines.xhtml

Keywords – Paediatrics, cardiac surgery, outcome, complications, morbidity, quality of life

Word count 3575

For peer review only

For peer review only - http://bmjopen.bmj.com/site/about/guidelines.xhtml

Abstract

Objectives:
Given the current excellent early mortality rates for paediatric cardiac surgery, stakeholders believe that this important safety outcome should be supplemented by a wider range of measures. Our objectives were to prospectively measure the incidence of morbidities following paediatric cardiac surgery and to evaluate their clinical and health-economic impact over 6-months.

Design:
The design was a prospective, multi-centre, multidisciplinary mixed methods study.

Setting:
The setting was five of the ten paediatric cardiac surgery centres in the United Kingdom with 21 months recruitment.

Participants:
The participants were children under the age of 17 years with heart diseases.

Results:
Families and clinicians prioritized:

Acute neurological event, unplanned re-intervention, feeding problems, renal replacement therapy, major adverse events, extracorporeal life support, necrotising enterocolitis, post-surgical infection, and prolonged pleural effusion or chylothorax.

Amongst 3090 consecutive surgeries there were 675 (21.8%) with at least one of these morbidities. Independent risk factors for morbidity included neonatal age, complex heart disease and prolonged cardiopulmonary bypass (P<0·001). Amongst patients with morbidity, 6-month survival was 88·2% (95% CI 85·4, 90·6) compared to 99·3% (95% CI 98·9, 99·6) with none of the morbidities (P<0·001).

Follow up for impact of morbidity amongst 666 patients over 6 months indicated that:

- Morbidity related impairment in health related quality of life improved between 6 weeks and 6 months.

For peer review only - http://bmjopen.bmj.com/site/about/guidelines.xhtml

- There were a median of 13 (95% CI (10.2, 15.8) P<0·001) fewer days at home with morbidity compare to no morbidity.
- Morbidities were costly: there was an adjusted incremental cost of £21,292, (95% CI £17,694, £32,423) P<0.001 with morbidity compared to no morbidity.

Conclusions:
Evaluation of post-operative morbidity is more complicated than measuring early mortality. However, tracking morbidity after paediatric cardiac surgery over 6 months offers stakeholders important data that is of value to parents and will be useful in driving future quality improvement.

For peer review only

Strengths and limitations of this study

- The study prospectively measured the incidence of defined morbidities that are considered to be important by both lay people and clinicians, in children undergoing heart surgery across five UK centres.
- Based on data collected, the study explored the risk factors for paediatric cardiac surgery morbidity statistically.
- Within a subset of patients, the study explored the impact of the selected morbidities on patients and families over 6 months, in terms of quality of life, days spent at home rather than in hospital and hospital costs.

Study registration

The study has ethical approval from London City Road Research Ethics Committee (14-LO-1442).

Funding details

This project was funded by the National Institute for Health Research Health Services and Delivery Research programme (Project No: 12/5005/06). The views and opinions expressed therein are those of the authors and do not necessarily reflect those of the NIHR HS&DR programme or the Department of Health. The funder had no role in the study.

Conflicts of interest

None declared by authors.

Data sharing

For data to be made available specific ethical and HRA approval would be required.

Background

Over 5000 paediatric cardiac surgery procedures are performed in the UK each year and early survival has improved to over 98% since comprehensive national audit commenced in 2000. [1] [2] Most stakeholders including clinicians, commissioners and users believe that whilst these early survival rates remain an important safety measure, it is has become imperative to explore a broader range of measures for outcome in this complex field of practice. Whilst there has been considerable research related to measuring, understanding and reducing peri-operative mortality for paediatric cardiac surgery, [3-5]much less attention has been focussed on surgical morbidities.

Morbidity is defined as a state of being unhealthy, or of experiencing an aspect of health that is 'generally bad for you'. In this project, by 'morbidity' we mean a defined aspect of ill health associated with a specific operation. In our study, we aimed to identify which morbidities present the greatest burden on patients and health services following paediatric cardiac surgery. Views may differ between professionals and non-professionals as to what the term morbidity exactly refers to and which surgical morbidities are most important. [6] Therefore we set out to combine patient and carers' perspectives with those of professional groups in defining a prioritised list of morbidities for prospective evaluation.

Over four years we:

1. Identified and defined nine morbidities following paediatric heart surgery, taking into account views from patients, carers, psychologists, nurses and clinicians, which together captured important aspects of the clinical and health-economic burden;
2. Measured incidence of the defined morbidities in the UK patient population and in subgroups defined by case complexity;
3. Evaluated the impact of defined morbidities on quality of life and estimated their clinical and health economic burden.

For peer review only - http://bmjopen.bmj.com/site/about/guidelines.xhtml

Methods

Design

The design was a prospective, multi-centre, multidisciplinary mixed methods study. Selection of morbidities was based on consensus methods. Morbidity incidence was evaluated with a prospective cross sectional study. Measurement of morbidity impact entailed a prospective, case-matched, longitudinal study.

Setting

Five UK paediatric cardiac surgery centres representing a range of programme sizes, which care for half of all patients nationally.

Participants

The participants were children under 17 years old with congenital heart disease (CHD).

Which morbidities?

Between January 2014 and September 2015, we reviewed existing literature, ran three family focus groups and undertook a family online discussion forum moderated by the Children's Heart Federation (a user group). Transcripts were thematically analysed and the resultant themes, together with the literature, helped to inform a long list of candidate surgical morbidities. A multi-disciplinary group, with patient and carer representation, then ranked and selected a list of nine key morbidities using the nominal group technique and secret voting.[7] This 'selection panel' [7] was informed in turn by clinical views on definitions and feasibility of routine monitoring for each candidate morbidity provided by an independent 'definition panel' [8] as reported in previous publications.

Incidence of morbidity

Between October 2015 and June 2017, we prospectively measured instances of morbidity within all consecutive surgical admissions across the study sites. Appendix 1 [8] provides the details of the criteria followed to define each individual morbidity, including the timeframe for diagnosis. Morbidities were attributed to the immediately preceding cardiac surgery and defined within the same hospitalisation. The only exceptions were unplanned re operation, which was defined as an unanticipated procedure within 30-days and mediastinitis, which could be identified post-discharge by the operating surgeon. [8] Data were regularly checked for completeness, clinical congruence and accuracy and, a three month sample of data was independently validated against the national audit data.

For peer review only - http://bmjopen.bmj.com/site/about/guidelines.xhtml

For peer review only

As for the UK audit of 30-day mortality, [9] all procedures more than 30 days apart on the same patient, were included in the morbidity analysis. As a secondary outcome, we checked each patient's survival status at 6 months after first appearance in the dataset, based on the individual centres National Congenital Heart Diseases Audit (NCHDA) [2] data and local hospital records.

Impact of morbidity

Recruitment

Recruitment to the impact study ran between October 2015 and June 2017 for all but one site, where it stopped after 6 months due to resource constraints. We attempted to recruit all patients with morbidity from the wider population, when at least one parent spoke reasonable English, and the family were resident in the UK. When feasible, for each morbidity case, we recruited a patient with no morbidity, matched on treating centre, age and univentricular status. The recruitment strategy led to the recruitment of a sample that was evenly balanced between patients with morbidity and those with no morbidity, however we were not able to find a match for every morbidity-patient. In order to account for the widest possible spectrum of important outcomes we included the available data on children who subsequently died.

Measures of impact

We evaluated the impact of morbidities over the 6 months following surgery, based on the following outcomes:

a) Quality of life and psychological burden on children and parents

We used age-specific measures at 6 weeks and 6 months following surgery. These were the PedsQL subscales (physical and psychosocial) and total scores. These scores range from 0 and 100, a higher score indicating better quality of life. PedsQL scores in a normal healthy population vary by age; expected scores for infants 0-12 months (which encompasses the median age for our cohort) are mean physical 84·98 (SD 9·45), mean psychosocial 80·47 (SD 12·64) and mean total 82·47 (SD 9·95). [10-12]

For the parents, we used the PHQ-4, which comprises four questions, measuring anxiety and depression in adults. Individual items are scored from zero to three. Scores ≥three for the first two questions suggest anxiety, and scores ≥three for last two questions suggest depression. [13] In the normal population, 4·8% have scores suggestive of anxiety and 6·6% have PHQ-4 scores suggestive of depression. [14]

For peer review only - http://bmjopen.bmj.com/site/about/guidelines.xhtml

b) Days at home over 6 months as an additional measure of disruption to family life

Length of inpatient stay following the index procedure was defined as the number of days between the operation and the date of discharge from the tertiary centre. Hospital stays that exceeded the 6-month follow-up period were censored after 183 days. All subsequent hospital admissions were captured and the total days spent in hospital including tertiary and secondary care admissions was subtracted from 183 days to generate a value for days at home by 6 months. Children who died in hospital were assigned a value of zero for this outcome.

c) Costs of the index hospitalisation

We calculated the cost of the inpatient stay in the tertiary centre following the index surgical procedure as a key indicator of the economic burden to the hospital provider (measured in 2016/17 UK£).

Costs were calculated as the summation of days in intensive care unit (ICU), days on the ward, and all operating procedures within the defined period. For ICU stays, data were recorded on the level of care the patient received each day (Enhanced Care, High Dependency, High Dependency Advanced, ICU Basic, ICU Basic Enhanced, ICU Advanced, ICU Advanced Enhanced and ECLS). [15] Unit costs of ICU stays were daily costs for each level of care (ranging from £870 to £5440) applied to the number of days spent receiving that level of care. Unit costs per day for ward stays were obtained from the highest recruiting centre and varied according to the age of the patient (<two years, £904; ≥two years, £680). Unit costs for surgical procedures were costs per minute supplied by study sites; these varied by site and so we used the value for the site that recruited the largest number of patients to this study (£13·39 per minute), which was similar to the mean value across all sites.

Statistical and Health Economic Analyses

Patient sample size

We anticipated that between 3000 and 3300 surgical patients would be admitted across the five sites during the study period. [2] We assumed that a clinically relevant difference in quality of life between matched pairs corresponded to a mean difference in quality of life score of at least 0.5 standard deviations. [16] To detect such a difference for PedsQL responses at 5% significance with 80% power requires a minimum of 32 matched pairs. Allowing for a 10% loss to follow-up rate, we aimed to recruit 36 matched pairs for each patient with morbidity, giving 80% power to detect a significant effect for any morbidity with a prevalence of at least 1·5%.

For peer review only - http://bmjopen.bmj.com/site/about/guidelines.xhtml

Analysis of risk factors for morbidity

Based on the whole sample of cardiac surgeries across the five sites over the study period, we explored risk factors for occurrence of morbidity. Clinical risk factor groups were derived from the finer diagnostic coding, based on previous peer reviewed research by our group. [9 17] We used multilevel logistic regression analysis to explore the role of pre-operative, patient-level case mix factors on the occurrence of any morbidity versus no morbidity, accounting for multiple procedures within patients. Firstly a univariate model to predict risk of any morbidity vs none was fitted for each risk factor. The estimated Odds ratios (OR) are presented along with 95% confidence intervals (CI). Then all factors significant on univariate analysis ($p<0 \cdot 1$) were considered in a multivariable model. We used multiple imputation by chained equations to account for missing data. The imputation model included all risk factors considered in the univariate analysis, which we assumed included all predictors of missingness. We indicate missing data in our results. The multivariable models were derived by fitting a regression model for all significant predictors and estimates were combined using Rubin's rules. [18]

Secondary outcome

The secondary outcome of survival at 6 months was calculated for all patients who had cardiac surgery at the sites within the study period. Unadjusted 6-month mortality rates were compared between morbidity groups using logistic regression.

Analysis of the impact of morbidity

In the patients recruited to the impact study, we analysed the 'impact outcomes' over 6 months post-surgery.

We used mixed effects regression models for PedsQL/PHQ-4 results to compare the impact of morbidity (any versus none) on outcome. All models were adjusted for clustering within matched pairs and significant covariates associated with incidence of morbidity. We used multiple imputation, by chained equations to account for missing data for those patients known to be alive at 6 weeks and 6 months respectively.

We used quantile regression to estimate the effects of individual morbidities in terms of differences in median days at home by 6 months, since the measure was negatively skewed. All models were adjusted for clustering within matched pairs and significant covariates associated with incidence of morbidity. Given the high level of data completeness, imputation was not used.

For peer review only - http://bmjopen.bmj.com/site/about/guidelines.xhtml

The inclusion of hospital site made negligible difference to either of these analyses, and not included in the statistical models.

We modelled the relationship between morbidities and cost of index hospitalisation, adjusted for significant covariates associated with hospital costs. Costs were skewed and to account for this we used a generalised linear model (GLM) with gamma family and log link, which has been recommended for modelling positively skewed data. [19] Missing outcome values were imputed using an iterative Markov chain Monte Carlo procedure based on multivariate normal regression, generating 20 imputed datasets. The hospital site was included as a significant covariate in the health economic model.

All analyses were performed in Stata 14. [20]

Patient and public involvement

The patient or user perspective lies at the core of the study methodology. Specifically, a key goal of the study was to consider the views of patients and parents when measures of morbidity are selected for future audit and benchmarking, in particular since emphasis may potentially differ between professionals and parents / patients.

Patient and family representatives from the Children's Heart Federation (CHF) were involved in aspects of the study design including design and running of the focus groups and the online discussion forum.

Representatives of the CHF participated in the facilitated nominal group meetings to select morbidities for inclusion in the incidence and impact studies.

A member of the CHF and a parent representative sat on the project steering group.

Results

1: Which morbidities?

Our final selected [7] and defined [8] list of included morbidities were:

Acute neurological event, unplanned re-intervention, feeding problems, renal replacement therapy, major adverse events, extracorporeal life support (ECLS), necrotising enterocolitis, post-surgical infection and prolonged pleural effusion or chylothorax.

We treated children with more than one of these events as a distinct group referred to as 'multi-morbidity'. Recognising ECLS as a very severe event, which nearly always occurs

For peer review only - http://bmjopen.bmj.com/site/about/guidelines.xhtml

with other morbidities, we treated ECLS as standalone morbidity (and always excluded from the multiple morbidity group). [21-25]

There was some divergence between the views of clinicians and families about the fundamental issue of what the important morbidities linked to paediatric cardiac surgery are. Health professionals from tertiary centres tended to prioritise clearly clinical issues related to the heart (e.g. use of ECLS and re operation) whereas parents placed greater emphasis on holistic outcomes for their child (e.g. feeding difficulties and child developmental problems). [7]

2. Incidence of morbidity

Descriptive results

We collected data on 3248 cardiac procedures and analysed 3090, after excluding 99 cardiac re-operations (a morbidity outcome) and 59 procedures that did not meet inclusion criteria.

The incidence of individual morbidities in isolation and overall for each is shown in Figure 1.

Figure 1 Incidence of morbidities

The figure shows the distinction between occurrence as a single morbidity and occurrence in combination with other morbidities, with 95% confidence intervals.

For peer review only

Significant risk factors for morbidity

As shown in Table 1, the most important risk factor for occurrence of 'any morbidity' was young age, particularly neonates, who were approximately five times as likely to experience a morbidity compared to children over one year old. Children with more complex heart disease or children whose operation included a bypass time longer than 90 minutes were almost twice as likely to experience morbidity. Other important independent risk factors were: palliative or staged procedures, the presence of functionally univentricular heart and pre-procedure severity of illness factors (e.g. pre-procedure mechanical ventilation or shock).

Table 1Summary of risk factors by any morbidity outcome

The weight was missing in 87 (2·8%) procedures and weight for age was set to missing for an additional 99 (3·2%) patients in whom the value was >5 SD from the normative mean. Other data were complete. Values include imputed data.

Cardiac diagnosis group A) hypoplastic left heart syndrome, Truncus arteriosus, pulmonary atresia intact septum, B) Functionally univentricular heart, pulmonary atresia ventricular septal defect, C) transposition of the great arteries all types, interrupted aortic arch, totally anomalous pulmonary venous connection, D) patent ductus arteriosus, tricuspid valve anomalies, acquired heart disease, complete atrioventricular septal defect, E) Tetralogy of Fallot, mitral valve anomalies, isolated aortic stenosis, aortic regurgitation, aortic arch obstruction, subaortic obstruction, ventricular septal defect, atrial septal defect.

The results of the multiple logistic regression model for occurrence of 'any morbidity', are expressed as odds ratio for 'any morbidity' by the stated factor, adjusted for age band, low weight, cardiac diagnostic category, functionally univentricular heart, acquired comorbidity, congenital comorbidity, severity of illness indicators, additional cardiac risk factors, specific procedure group, bypass time category.

	None of the morbidities (N = 2415)	Any Morbidity (N=675)	Univariate odds ratio for 'any morbidity' (95% CI), P Value	Multivariable odds ratio for 'any morbidity' (95% CI), P Value
**Male**	1299 (53.8)	372 (55.1)	1.05 (0.89, 1.25), P=0.54	-
**Median Age (days) (IQR)**	286 (105, 1582)	102 (10, 331)		
**Child**	1111 (46.0)	160 (23.7)		
**Infant**	1023 (42.4)	268 (39.7)	1.82 (1.47, 2.25), P<0.001	1.61 (1.26, 2.05), P<0.001
**Neonate**	281 (11.6)	247 (36.6)	6.10 (4.81, 7.75)< P<0.001	5.26 (3.90, 7.09), P<0.001
**Median Weight (Kg) (IQR)**	7.7 (4.7, 16.2)	4.6 (3.2, 8.0)		
**Low weight <2 SD below mean**	714 (31.5)	234 (36.8)	1.27 (1.05, 1.52)·P=0.01	1.21 (0.97, 1.51), P=0.09
**Cardiac Diagnosis Group**				
**E Least complex (reference)**	1002 (41.5)	123 (18.2)		
**D**	796 (33.0)	227 (33.6)	5.13 (3.79, 6.93), P<0.001	2.02 (1.58, 2.60), P<0.001
**C**	215 (8.9)	109 (16.2)	3.83 (2.85, 5.13), P<0.001	1.44 (1.00, 2.07), P=0.05
**B**	232 (9.6)	109 (16.2)	4.13 (3.07, 5.55), P<0.001	2.62 (1.85, 3.71), P<0.001
**A Most Complex**	170 (7.0)	107 (15.8)	2.32 (1.83, 2.94), P<0.001	2.14 (1.41, 3.24), P<0.001
**Functionally univentricular heart**	255 (10.6)	159 (23.6)	2.61 (2.11, 3.23), P<0.001	1.55 (1.07, 2.24), P=0.02
**Acquired comorbidity**	337 (14.0)	119 (17.6)	1.32 (1.05, 1.66), P=0.02	1.33 (1.03, 1.71), P=0.03
**Congenital comorbidity**	537 (22.2)	178 (26.4)	1.25 (1.03, 1.52), P=0.03	1.28 (1.02, 1.59), P=0.03
**Severity of illness indicators**	222 (9.2)	152 (22.5)	2.87 (2.30, 3.58), P<0.001	1.52 (1.16, 2.00), P<0.01
**Premature <37 weeks gestation**	231 (9.6)	73 (10.8)	1.15 (0.87, 1.51), P=0.33	-
**Downs Syndrome**	214 (8.9)	63 (9.3)	1.06 (0.79, 1.43), P=0.71	-
**Additional cardiac risk**	165 (6.8)	65 (9.6)	1.45 (1.09, 1.94), P=0.01	1.39 (0.99, 1.94), P=0.05
**Surgical procedure type**				
**Reparative/corrective (reference)**	1391 (57.6)	332 (49.2)		
**Palliative/staged**	331 (13.7)	179 (26.5)	2.27 (1.82, 2.82), P<0.001	1.65 (1.14, 2.38), P<0.01
**Ungrouped**	693 (28.7)	164 (24.3)	0.99 (0.81, 1.22), P=0.94	1.04 (0.82, 1.31), P=0.75
**Median bypass time (mins) (IQR)**	72 (42, 110)	110 (62, 156)		
**No bypass (reference)**	390 (16.2)	103 (15.3)		
**Up to 90 mins**	1148 (47.5)	150 (22.2)	0.48 (0.35, 0.65), P<0.001	0.78 (0.57, 1.09), P=0.14
**More than 90 mins**	877 (36.3)	422 (62.5)	1.76 (1.32, 2.34), P<0.001	2.28 (1.67, 3.12), P<0.001

For peer review only - http://bmjopen.bmj.com/site/about/guidelines.xhtml

Secondary outcomes

Patient survival was highest and patient hospital stay was shortest for patients with none of the morbidities (see Table 2). The 6-month survival was significantly lower for patients with a single morbidity, for those with ECLS and multiple morbidities, compared with patients with none of the morbidities $P < 0{\cdot}001$ for all.

Table 2 Secondary outcomes of hospital stay and survival at 6 months by morbidity

The number (%) of morbidities was based on individual surgical episode, as was the length of stay, whereas survival was calculated per patient. Data are presented based on morbidity in isolation, multi-morbidity and ECLS. Length of stay data was missing for 9 patients and Life status was missing in 16 patients who were not included.

For peer review only

Morbidity type	Number (%)	Median length of post-operative hospital stay in days (IQR)	6 month survival Number, %, (95% CI)
**No morbidity**	2415 (78.2%)	8 days (IQR 5, 13)	2202/2217 99.3% (98.9, 99.6)
**Any morbidity**	675 (21.8%)	24 days	675 (21.8%)
**Acute neurological event**	14 (0.5)	19 days (12, 39)	12/13 92.3% (64.0, 99.8)
**Unplanned re-intervention**	59 (1.9%)	22 days (14, 33)	50/54 92.6% (82.1, 97.9)
**Feeding problems**	99 (3.2%)	20.5 days (12, 36)	90/91 98.9% (94, 100)
**Renal support**	40 (1.3%)	17 days (14, 26)	37/39 94.9% (82.7, 99.4)
**Major adverse event**	34 (1.1)	16.5 days (8, 25)	28/33 84.9% (68.1, 94.9)
**Necrotising enterocolitis**	32 (1.0%)	24.5 days (18.5, 49.5)	28/30 93.3% (77.9, 99.2)
**Post-surgical infection**	27 (0.9%)	20.5 days (11, 28)	25/25 100% (86.3, 100)
**Prolonged pleural effusion**	111 (3.6%)	20 days (14, 28)	95/96 99.0% (94.3, 100)
**Multi- morbidity**	197 (6.4)	35 days (IQR 22, 56)	158/190 83.2% (77.1, 88.2)
**Extracorporeal life support**	62 (2.0%)	43 days (IQR 20, 84)	31/57 54.4% (40.7, 67.6)

3. Impact of morbidity

Patient sample

We recruited 60% of eligible families, amounting to 666 children of whom 340 (51%) had at least one morbidity. Of these, 19 died within 30 days and 39 died within 6 months. We pair matched 558 patients and 108 patients unmatched of whom 61 had morbidity; all of the patients were included in the statistical analysis.

Quality of life and anxiety - depression outcomes

For the 6-weeks data, a comparison between patients with missing versus non missing data indicated the only difference was in the proportions with severity of illness factors and

For peer review only - http://bmjopen.bmj.com/site/about/guidelines.xhtml

additional cardiac risk factors. Importantly there was no difference in the proportion of patients with or without morbidity. At the 6 months mark there was no difference in any baseline factor or morbidity occurrence.

All PedQL scores were lower than in a healthy population (see methods [10-12] and Table 3). In terms of our study objective to assess the impact of morbidity; the total PedsQL scores were significantly reduced for children with any morbidity at 6 weeks in comparison to children with none of the morbidities (case mix adjusted score reduction of -5·2 (95% CI -8·3, -2·2) P<0·01). On a positive note, this difference narrowed to small non-significant reduction in total score by 6 months. Physical scores were affected more than psychosocial scores, and were still significantly impaired at 6-months.

All parents experienced higher rates of both anxiety and depression than a healthy population (see methods and Table 3). The parents of children with morbidity were around 57% more likely to experience anxiety; and around 77% more likely to experience depression, at 6 weeks post operation than parents of children without a morbidity. PHQ-4 scores had improved by 6 months, and although there remained a higher chance of both anxiety and depression with morbidity, the difference narrowed and was not significant.

For peer review only - http://bmjopen.bmj.com/site/about/guidelines.xhtml

Table 3: Quality of life and parental anxiety / depression based on whether or not a morbidity was present

Quality of life (QOL) was derived from PedsQL scores for 478 children at 6 weeks (26% missing) and 403 children at 6 months (36% missing).

Parental anxiety and depression was derived from PHQ-4 responses of 481 parents at 6 weeks (26% missing) and 394 parents at 6 months (37% missing).

Values include imputed data.

The differences in each outcome are adjusted for age band, low weight, cardiac diagnostic category, functionally univentricular heart, acquired comorbidity, congenital comorbidity, severity of illness indicators, additional cardiac risk factors, specific procedure group, bypass time category.

For peer review only

The impact outcome type and time point of measurement	Patients with none of the morbidities	Patients with any morbidity	Differences in outcome based on presence of any morbidity
	Score Mean (SD)	*Score Mean (SD)*	*Adjusted difference in mean score with morbidity (95% CI), P value*
**Physical QOL Score at 6 weeks**	79.0 (16.2)	69.1 (21.8)	-8.3 (-11.8, -4.9), P<0.001
**Psychosocial QOL Score at 6 weeks**	79.4 (14.7)	75.2 (19.0)	-2.7 (-5.9, 0.5), P=0.08
**Total QOL Score at 6 weeks**	79.3 (13.8)	72.1 (19.1)	-5.2 (-8.3, -2.2), P<0.01
	Score Mean (SD)	*Score Mean (SD)*	*Adjusted difference in mean score with morbidity* (95% CI), P value
**Physical QOL Score at 6 months**	82.3 (16.6)	76.6 (18.6)	-4.2 (-7.6, -0.8), P=0.02
**Psychosocial QOL Score at 6 months**	78.0 (14.7)	76.4 (15.4)	-0.9 (-3.5, 1.8), P=0.5
**Total QOL Score at 6 months**	79.8 (13.9)	76.6 (15.0)	-2.3 (-4.9, 0.3), P=0.08
	Number (%) with attribute	*Number (%) with attribute*	*Adjusted odds ratio for outcome by morbidity* (95% CI)
**Parental Anxiety at 6 weeks**	55 (23.7)	92 (36.8)	1.57 (1.04, 2.35), P=0.03
**Parental Depression at 6 weeks**	26 (11.2)	55 (22.1)	1.77 (1.10, 2.86), P=0.02
	Number (%) with attribute	*Number (%) with attribute*	*Adjusted odds ratio for outcome by morbidity (95% CI)*
**Parental Anxiety at 6 months**	23 (11.5)	34 (17.4)	1.37 (0.77, 2.43), P=0.28
**Parental Depression at 6 months**	15 (7.5)	23 (11.9)	1.45 (0.79, 2.68), P=0.23

Days at home by 6 months outcome

Children with none of the morbidities had a median of 174 days at home (IQR 169, 176) out of a possible 183 days in 6 months. The presence of any morbidity reduced the median days at home by 13 days over 6 months (95% CI (10·2, 15·8) P<0·001). When individual morbidities were each considered in turn, the difference in the case mix adjusted median days at home was significantly lower at 6 months for all of them apart from 'renal support' (P<0.05).

Health economic outcome

The costs of the index hospitalisation were available for 613 patients (8% missing). The mean additional cost linked to having any of the selected morbidities was £21,292, (95% CI £17,694, £32,423). P<0.001. In Table 4, we present the adjusted marginal effects, which are

For peer review only - http://bmjopen.bmj.com/site/about/guidelines.xhtml

the adjusted incremental costs linked to the stated morbidity, in comparison to the adjusted cost linked to patients with none of the selected morbidities. The greatest adjusted cost difference was for ECLS, where the adjusted mean difference in costs was £62,452; followed by multi-morbidity, where the adjusted mean difference was £33,147 (P<0·01 for both). Significantly increased (P<0·05) costs were also found for unplanned re-intervention, feeding problems, renal support and prolonged pleural effusion. The remaining morbidities, representing the lowest number of patients per category, showed non-significant differences.

Table 4 Cost of hospital stay following index surgical procedure by morbidity

Figures in the Marginal Effect column are the mean difference in costs between each morbidity category and the category of no selected morbidities present, conditional on the covariates. Costs are in 2016/17 UK£.

Values include imputed data.

The marginal effects are adjusted for age band; weight-by-age z score; congenital morbidity, severity of illness indicator, Downs' syndrome; cardiac diagnosis category; cardiac procedure category; whether or not the patient died within 6 months; and, study site.

**Morbidity**	**Coef.**	**Marginal Effect (£) (95% CI), P Value**
**Any morbidity (n=340)**	0.71	21,292, (95% CI 17,694, 32,423). P<0.001.
**Acute neurological event (n=6)**	0.50	13,911 (-3,923, 50,059), P=0.16
**Unplanned re-intervention (n=26)**	0.62	18,330 (7,475, 33,282), P<0.01
**Feeding morbidity (n=45)**	0.55	15,541 (7,200, 26,326), P<0.01
**Renal support (n=24)**	0.41	10,798 (1,846, 23,215), P=0.01
**Major adverse event (n=22)**	0.30	7,555 (-1,131, 19,984), P=0.10
**Necrotising enterocolitis (n=11)**	0.37	9,547 (-2,173, 28,454), P=0.13
**Post-surgical infection (n=11)**	0.27	6,466 (-3,947, 23,130), P=0.17
**Prolonged pleural effusion (n=50)**	0.33	8,177 (1,444, 16,904), P=0.01
**Extracorporeal life support (n=27)**	1.37	62,452 (39,546, 93,983), P<0.01
**Multi-morbidity (n=118)**	0.94	33,147 (23,669, 44,624)· P<0.01

Discussion

Overview of findings

This paper presents the results of the first prospective multi-centre study of morbidity after paediatric cardiac surgery morbidity that measured outcome 6-months after operation. The results are novel and indicate that evaluation of morbidity adds considerably to the current main metric of 30-day or in hospital survival.

For peer review only - http://bmjopen.bmj.com/site/about/guidelines.xhtml

We note the excellent outcomes in children who had none of the morbidities: their survival at 6-months post-operation was 99·3% and their median length of stay was 8 days, suggesting that the morbidities we selected do capture most of the complication-related adverse outcomes for this context.

Based on our impact sub study, occurrence of ECLS and multiple morbidities are particularly important adverse outcomes – they are associated with high mortality, high resource use and lower quality of life for surviving patients over 6 months after surgery. Future routine monitoring and public reporting for these should be considered by centres and the national audit.

In our engagement work, patients' families consistently told us that outcomes affecting the child as a whole over the medium or longer term are very important to them. We thus included morbidities such as feeding problems and acute neurological events but note that those types of outcome were the most complicated to capture, and we intend to publish additional material on these topics.

Limitations

We acknowledge that the definition, measurement, reporting and interpretation of morbidity, is much more complex than for mortality. This activity requires significant staff resources and clinician buy in, in particular because some morbidities are challenging to measure and to interpret. The number of individual morbidities was low in some of our analyses, limiting interpretation. We have not captured every single morbidity that exists after children's heart surgery.

Context

There have been previous attempts at reporting morbidity after paediatric cardiac surgery, however these have in general captured events within a hospital setting, whereas our study captures outcomes out to 6 months post-surgery. Two single-centre studies generated an aggregate 'Morbidity Index' by assigning subjective weights to post-operative complications. [26 27] The Society of Thoracic Surgery in the USA, developed a 'Morbidity Score', based on data from their multi-centre registry. [28] We note that condensing diverse morbidities into a single score may lead to loss of information. Moreover, recent work on using graphical methods to routinely monitor a range of morbidities [29] highlighted the complexity of graphically summarising multiple morbidities. [30] The Pediatric Cardiac Critical Care Consortium (PC^4) was set up in 2009, and provides partner sites with access to real-time,

For peer review only - http://bmjopen.bmj.com/site/about/guidelines.xhtml

reliable and actionable data to be used for local quality improvements. [31-34] However membership of PC[4] is voluntary for institutions on a subscription basis, the reported measures been selected by clinicians, the reporting of outcomes is accessible only to subscribing member institutions and these are limited to in hospital measures.

Future practice and research

Neurodevelopmental problems are common in children with CHD undergoing surgery [35] however we detected acute neurological events (ANE) following only 2·1% of procedures. : Therefore it appears that peri-operative 'ANE' represents the tip of the iceberg, and hence the scope of surveillance for child neurodevelopment in CHD needs to extend well beyond the peri-operative period.

Despite their great importance to families, we note that participants reported subjectivity in the collection of data on 'feeding problems'; hence these were difficult to capture consistently. Additional research may help determine the best way to measure this important morbidity and to elucidate approaches to alleviate the impact of feeding problems in CHD.

Our measure of renal failure was the need for renal support, and although relatively easy data to collect this may not be the optimal method to capture this morbidity, given that there are differences in practice between clinicians. [36] Further research may help us to understand the best approach to manage post-operative renal injury in CHD.

Our results perhaps unsurprisingly indicate that parents of children who suffer morbidities experience higher rates of anxiety and depression post-operatively. This emphasises the importance of supporting parents during this phase.

We used study data to co-develop parent and carer information resources, showing what the morbidities are and how their incidence and the length of stay may vary based on the complexity of the child's condition. Parents told us that it helps to know that; firstly they are not alone in facing a morbidity, secondly clinical teams have seen morbidities before and know how to deal with them, and thirdly that it is better as a parent to 'be prepared'. Furthermore, parents indicated that information about impact such as nearly all children who experience a morbidity and recover will have a similar quality of life to children who did not experience a morbidity by the six month mark, was very helpful to know.

Within the scope of this project, a new Excel tool has been developed and piloted, which enables clinical teams to benchmark and report the local rates of morbidities with a quality

For peer review only - http://bmjopen.bmj.com/site/about/guidelines.xhtml

For peer review only

assurance goal, such as in a mortality and morbidity conference. Furthermore, we have kept in close contact with the National Congenital Heart Diseases Audit (NCHDA) and the Clinical Reference Group for CHD services. NCHDA has already started to collect five of the nine morbidities within the nationally mandated dataset, using the definitions that we developed.

For peer review only

Author contributions

Designed the study and wrote the protocol: Katherine Brown, Christina Pagel, Martin Utley, Deborah Ridout, Jo Wray, Steve Morris, and Victor Tsang.

Clinical expertise: Victor Tsang, David Anderson, David Barron, Serban Stoica, Andrew Maclean, Katherine Brown, Shane Tibby, David Barron, Peter Davis and Warren Rodrigues.

Study implementation, protocol adjustments, data cleaning: Katherine Brown, Alison Jones, Karen Sheehan, Victor Tsang, Jane Cassidy, David Barron, Peter Davis, Serban Stoica, Warren Rodrigues, and Shane Tibby.

Statistical analysis: Deborah Ridout, Christina Pagel, Shane Tibby, and Katherine Brown.

Health economic analysis: Emma Hudson, Steve Morris.

Contributed to the write up and signed off the paper: Katherine Brown, Christina Pagel, Deborah Ridout, Jo Wray, Victor Tsang, David Anderson, David Barron, Jane Cassidy, Peter Davis, Emma Hudson, Alison Jones, Andrew McLean, Steve Morris, Warren Rodrigues, Karen Sheehan, Serban Stoica, Shane Tibby, and Thomas Witter.

Acknowledgements (includes patient and public contributor names)

We would like to thank Martin Utley, Sheryl Snowball, Luke Maidment, Sarah Bohannon, Liz Smith, Kate Penny-Thomas, Joanne Webb, Sinead Cummins, John Stickley, Natasha Khan, Teresa Dickson, Ray Samson, Isobel Mcleod, Paul Wellman, Rhian Lakhani, Kathleen Selway, Carrie Cherrington, Andrew Parry, Rob Tulloh, Bill Gaynor, Rodney Franklin, Lisa Allera, Kate Bull, Trevor Ritchens, Branko Mimic, Jon Smith, Lyvonne Tume, Vibeke Hjortdal, Michael Vath, Tom Treasure, Anne Keatley Clarke, Bea Tuten, and all the patients who participated in the study.

For peer review only

For peer review only - http://bmjopen.bmj.com/site/about/guidelines.xhtml

References

1. Brown KL, Crowe S, Franklin R, et al. Trends in 30-day mortality rate and case mix for paediatric cardiac surgery in the UK between 2000 and 2010. *Open Heart* 2015;2(1):e000157. doi: 10.1136/openhrt-2014-000157 [published Online First: 2015/04/22]
2. NICOR. NICOR: National Institute for Cardiovascular Outcomes Research: Congenital Heart Diseases Website UCL London UK: University College London; 2017 [Available from: www.nicor4.nicor.org.uk/CHD accessed Jan 2017 2017.
3. O'Brien SM, Clarke DR, Jacobs JP, et al. An empirically based tool for analyzing mortality associated with congenital heart surgery. *J Thorac Cardiovasc Surg* 2009;138(5):1139-53. doi: S0022-5223(09)01017-4 [pii]

10.1016/j.jtcvs.2009.03.071 [published Online First: 2009/10/20]
4. Jenkins KJ, Gauvreau K, Newburger JW, et al. Consensus-based method for risk adjustment for surgery for congenital heart disease. *J Thorac Cardiovasc Surg* 2002;123(1):110-8. doi: S0022522302909451 [pii] [published Online First: 2002/01/10]
5. Lacour-Gayet F, Clarke D, Jacobs J, et al. The Aristotle score for congenital heart surgery. *Semin Thorac Cardiovasc Surg Pediatr Card Surg Annu* 2004;7:185-91. [published Online First: 2004/07/31]
6. Cooke M, Thackray S. Differences between community professional and patient perceptions of chronic obstructive pulmonary disease treatment outcomes: a qualitative study. *J Clin Nurs* 2012;21(11-12):1524-33. doi: 10.1111/j.1365-2702.2012.04094.x [published Online First: 2012/05/19]
7. Pagel C, Brown KL, McLeod I, et al. Selection by a panel of clinicians and family representatives of important early morbidities associated with paediatric cardiac surgery suitable for routine monitoring using the nominal group technique and a robust voting process. *BMJ open* 2017;7(5):e014743. doi: 10.1136/bmjopen-2016-014743 [published Online First: 2017/05/31]
8. Brown KL, Pagel C, Brimmell R, et al. Definition of important early morbidities related to paediatric cardiac surgery. *Cardiol Young* 2017;27(4):747-56. doi: 10.1017/S1047951116001256 [published Online First: 2016/09/30]
9. Rogers L, Brown KL, Franklin RC, et al. Improving Risk Adjustment for Mortality After Pediatric Cardiac Surgery: The UK PRAiS2 Model. *Ann Thorac Surg* 2017;104(1):211-19. doi: 10.1016/j.athoracsur.2016.12.014 [published Online First: 2017/03/21]
10. Varni JW, Seid M, Rode CA. The PedsQL: measurement model for the pediatric quality of life inventory. *Med Care* 1999;37(2):126-39. [published Online First: 1999/02/19]
11. Varni JW, Seid M, Kurtin PS. PedsQL 4.0: reliability and validity of the Pediatric Quality of Life Inventory version 4.0 generic core scales in healthy and patient populations. *Med Care* 2001;39(8):800-12. [published Online First: 2001/07/27]
12. Varni JW, Limbers CA, Neighbors K, et al. The PedsQL Infant Scales: feasibility, internal consistency reliability, and validity in healthy and ill infants. *Qual Life Res* 2011;20(1):45-55. doi: 10.1007/s11136-010-9730-5 [published Online First: 2010/08/24]
13. Kroenke K, Spitzer RL, Williams JB, et al. An ultra-brief screening scale for anxiety and depression: the PHQ-4. *Psychosomatics* 2009;50(6):613-21. doi: 10.1176/appi.psy.50.6.613 [published Online First: 2009/12/10]
14. Lowe B, Wahl I, Rose M, et al. A 4-item measure of depression and anxiety: validation and standardization of the Patient Health Questionnaire-4 (PHQ-4) in the general population. *Journal of affective disorders* 2010;122(1-2):86-95. doi: 10.1016/j.jad.2009.06.019 [published Online First: 2009/07/21]

For peer review only - http://bmjopen.bmj.com/site/about/guidelines.xhtml

15. https://www.picanet.org.uk/wp-content/uploads/sites/25/2018/05/PICANet_National_Report_2007.pdf. PCCMDS Leeds: HQIP; 2018 [accessed Jan 5 2018.
16. Norman GR, Sloan JA, Wyrwich KW. Interpretation of changes in health-related quality of life: the remarkable universality of half a standard deviation. *Med Care* 2003;41(5):582-92. doi: 10.1097/01.MLR.0000062554.74615.4C [published Online First: 2003/04/30]
17. Brown KL, Rogers L, Barron DJ, et al. Incorporating Comorbidity Within Risk Adjustment for UK Pediatric Cardiac Surgery. *Ann Thorac Surg* 2017;104(1):220-26. doi: 10.1016/j.athoracsur.2016.12.013 [published Online First: 2017/03/21]
18. Rubin DB. Multiple Imputation for Nonresponse in Surveys. In: Wiley, ed. New York, 1987.
19. Barber J, Thompson S. Multiple regression of cost data: use of generalised linear models. *Journal of health services research & policy* 2004;9(4):197-204. doi: 10.1258/1355819042250249 [published Online First: 2004/10/29]
20. 2015. S. Stata Statistical Software: Release 14. . In: LP. S, ed. College Station, TX:, 2015.
21. Aharon AS, Drinkwater DC, Jr., Churchwell KB, et al. Extracorporeal membrane oxygenation in children after repair of congenital cardiac lesions. *Annals of Thoracic Surgery* 2001;72(6):2095-101; discussion 101-2.
22. Alsoufi B, Shen I, Karamlou T, et al. Extracorporeal life support in neonates, infants, and children after repair of congenital heart disease: modern era results in a single institution. *Annals of Thoracic Surgery* 2005;80(1):15-21; discussion 21.
23. Beiras-Fernandez A, Deutsch M-A, Kainzinger S, et al. Extracorporeal membrane oxygenation in 108 patients with low cardiac output - a single-center experience. *International Journal of Artificial Organs* 2011;34(4):365-73.
24. Chauhan S, Malik M, Malik V, et al. Extra corporeal membrane oxygenation after pediatric cardiac surgery: a 10 year experience. *Annals of Cardiac Anaesthesia* 2011;14(1):19-24.
25. Coskun KO, Coskun ST, Popov AF, et al. Extracorporeal life support in pediatric cardiac dysfunction. *Journal Of Cardiothoracic Surgery* 2010;5:112.
26. Bojan M, Gerelli S, Gioanni S, et al. Evaluation of a new tool for morbidity assessment in congenital cardiac surgery. *Ann Thorac Surg* 2011;92(6):2200-4. doi: 10.1016/j.athoracsur.2011.08.017 [published Online First: 2011/11/26]
27. Costello JM, Cooper DS, Jacobs JP, et al. Intermediate-term outcomes after paediatric cardiac extracorporeal membrane oxygenation--what is known (and unknown). *Cardiol Young* 2011;21 Suppl 2:118-23. doi: 10.1017/S1047951111001697 [published Online First: 2011/12/14]
28. Jacobs ML, O'Brien SM, Jacobs JP, et al. An empirically based tool for analyzing morbidity associated with operations for congenital heart disease. *J Thorac Cardiovasc Surg* 2012 doi: 10.1016/j.jtcvs.2012.06.029 [published Online First: 2012/07/28]
29. Salvin JW, Scheurer MA, Laussen PC, et al. Blood transfusion after pediatric cardiac surgery is associated with prolonged hospital stay. *Ann Thorac Surg* 2011;91(1):204-10. doi: 10.1016/j.athoracsur.2010.07.037 [published Online First: 2010/12/22]
30. Utley M, Brown K, Tsang V. Invited commentary. *Ann Thorac Surg* 2012;94(5):1602-3. doi: 10.1016/j.athoracsur.2012.07.003 [published Online First: 2012/10/27]
31. Alten JA, Rahman AF, Zaccagni HJ, et al. The Epidemiology of Health-Care Associated Infections in Pediatric Cardiac Intensive Care Units. *Pediatr Infect Dis J* 2017 doi: 10.1097/INF.0000000000001884 [published Online First: 2017/12/28]
32. Hickey PA, Connor JA, Cherian KM, et al. International quality improvement initiatives. *Cardiol Young* 2017;27(S6):S61-S68. doi: 10.1017/S1047951117002633 [published Online First: 2017/12/05]
33. Gaies M, Werho DK, Zhang W, et al. Duration of Postoperative Mechanical Ventilation as a Quality Metric for Pediatric Cardiac Surgical Programs. *Ann Thorac Surg* 2018;105(2):615-21. doi: 10.1016/j.athoracsur.2017.06.027 [published Online First: 2017/10/11]

For peer review only - http://bmjopen.bmj.com/site/about/guidelines.xhtml

34. Buckley JR, Graham EM, Gaies M, et al. Clinical epidemiology and centre variation in chylothorax rates after cardiac surgery in children: a report from the Pediatric Cardiac Critical Care Consortium. *Cardiol Young* 2017:1-8. doi: 10.1017/S104795111700097X [published Online First: 2017/05/30]
35. Marino BS, Lipkin PH, Newburger JW, et al. Neurodevelopmental outcomes in children with congenital heart disease: evaluation and management: a scientific statement from the American Heart Association. *Circulation* 2012;126(9):1143-72.
36. Kwiatkowski DM, Goldstein SL, Cooper DS, et al. Peritoneal Dialysis vs Furosemide for Prevention of Fluid Overload in Infants After Cardiac Surgery: A Randomized Clinical Trial. *JAMA pediatrics* 2017;171(4):357-64. doi: 10.1001/jamapediatrics.2016.4538 [published Online First: 2017/02/28]

For peer review only

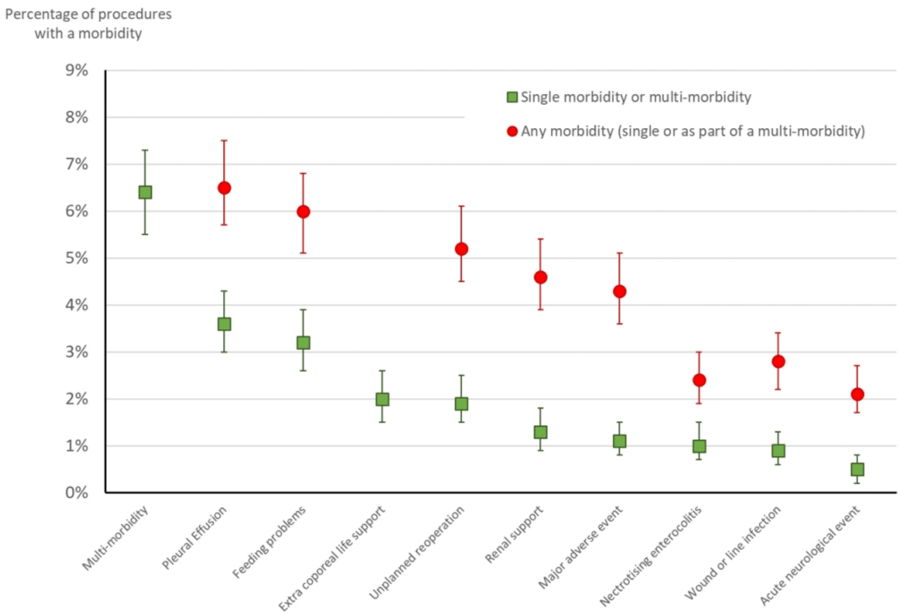

Figure 1 Incidence of morbidities
The figure shows the distinction between occurrence as a single morbidity and occurrence in combination with other morbidities, with 95% confidence intervals.

Appendix 1 from Brown KL, Pagel C, Brimmell R*, et al.* Definition of important early morbidities related to paediatric cardiac surgery. *Cardiol Young*. 2017;**27**:747-56 doi: 10.1017/S1047951116001256 [published Online First: 2016/09/30].

Morbidity	Timescale for identification	Definition	Measurement protocol (if additional to definition)	Minimum treatment protocol
**Acute Neurological Event**	Includes neurological morbidities that, based on best clinical judgement, arose as *new* findings around the time of surgery that were detected within the same hospitalisation as the surgery. It is recognised that in certain circumstances such as where a child is very sick on life support, pre procedure assessment is challenging, in these circumstances as full an evaluation as possible to be completed, incorporating serial assessments over time.	Neurological events including: seizure, abnormal movement (includes choreiform or athetoid), focal neurological deficit (includes hemiplegia and monoplegia), intracranial haemorrhage, stroke, brain death, Reversible Ischaemic Neurological Dysfunction, Hypoxic Ischaemic Encephalopathy, spinal cord ischaemia, basal ganglia damage or brain stem injury (includes abnormal cough or gag reflex). (1)	Includes new abnormality in any of the following:<br>- Electroencephalogram.<br>- Brain scan (either computerised tomography or magnetic resonance).<br>- Clinical evaluation<br>(Seizures or movement disorder, focal neurological signs, generalised neurological signs, altered conscious level including even brain death.)	The treatment protocol is variable depending on the type of neuro-morbidity.<br>Specialist consultation with a neurologist, a full evaluation of any brain injury and neuro-developmental follow up would be a minimum.

For peer review only

**Unplanned reoperation or re-intervention**	Unplanned re-interventions are procedures outside the expected patient pathway, which may be undertaken at any time from the start of the post-operative admission up until 30 days following the primary operation. Additional procedures or revisions undertaken within the primary trip to the operating theatre (incorporating return onto cardiopulmonary bypass) are not included in the definition of re-operation.	Unplanned re-interventions include procedures that were not intended during the planning phase, follow an initial primary cardiac surgery and result in "substantive alteration to heart" incorporating cardiac bypass, cardiac non bypass, pacemaker placement, interventional catheterisations and also diaphragm plications (which are not related to the heart itself). The definition does not include support or other non-cardiac surgery procedures.	Unplanned return to the operating room or cardiac catheter laboratory within 30 days<br>(Excludes interventional catheters that were planned pre-operatively; excluding delayed chest closure, excluding procedures for bleeding)<br>(Includes diaphragm plication and insertion of pace maker for surgically acquired arrhythmia).	Not applicable. The minimal assessment is cardiovascular evaluation of the repair with echocardiography and tolerance of weaning from life supports.
**Feeding problems**	A diagnosis of post-operative feeding problems should be considered during recovery after surgery and prior to discharge from the specialist centre either to home or to secondary care if	A child may fail to feed normally following paediatric cardiac surgery for a range of reasons including gastro-oesophageal reflux, vocal cord paralysis, oral-motor dysfunction, oral aversion, and neurologic impairment. (2) If for any of these reasons a child is not able to orally	The requirement for any feeding support.<br>Includes via the intravenous route or via an enteral tube.	Treatment includes assessment by the dietician, speech and language therapist and of the patient's weight. Progress with

	the child is unable to feed normally. The goal is detection of feeding problems which are new post-surgery, and it is recognised that this may be challenging where a child was not fed pre-operatively for cardiac reasons since feeding ability will not have been assessed objectively.	feed or completely orally feed and is tube dependent at discharge from the tertiary centre or at 30 days (if he or she is otherwise clinically stable enough to feed at that time point), then a post-operative feeding problem will be diagnosed.	Excludes feeding support that was present to treat a primary problem diagnosed before the surgery, feeding support related to an episode of necrotising enterocolitis, and feeding support because the child dislikes a special diet.	feeding should be monitored by the clinical care team responsible at each stage of the journey.
**Need for renal replacement therapy**	Includes renal replacement therapy when initiated as a new support at any time from the start of the post-operative admission to ICU up until 30 days following the primary operation.	The child requires renal replacement therapy (peritoneal dialysis or haemofiltration) for renal failure (oligo-anuria of less than 0.5 ml/kg/hour and elevated creatinine level for age) and or fluid overload. In patients where renal support is required alongside extracorporeal life support, the primary morbidity is viewed as extracorporeal life support.	The measurement protocol is simply the presence of (new) renal support. (Excludes renal support on extracorporeal life support). Data on renal biochemistry and urine output will be collected.	Instigation of effective renal replacement therapy.<br>If recovery of kidney function does not occur within 3 to 5 weeks then consultation with paediatric renal

For peer review only

				physician is required.
**Major adverse cardiac events or never events**	Events within this morbidity may be identified during the tertiary hospital stay (either ward or ICU) following the primary surgery.	These morbidity includes:<br>- Cardiac arrest, where the child receives any chest compressions or defibrillation.<br>- Chest re-opening on the ICU or ward for any reason.<br>- Major haemorrhage in the ICU following surgery.<br>- A 'Never Event' applicable to paediatric cardiac surgery as selected from the 'Never Events' list published for NHS for 2015 (3)<br>(Including wrong site or wrong patient surgery, wrong prosthesis surgery, retained foreign object post procedure, wrong route administration of medication, transfusion or transplantation of main red cell group incompatible blood components or organs, misplaced naso-gastric or oro-gastric tubes,	Major haemorrhage is defined as bleeding > 10ml/kg/hr on ICU for 2 consecutive hours.<br>A 'Never Event' includes the events listed plus harm to the patient, for example: if a naso-gastric tube is misplaced, detected and removed in a timely manner before any harm is done then this is not a 'Never Event'. Conversely, if the misplaced naso-gastric tube is not noted, and feed is given into the bronchus, then this is a 'Never Event'.	All events will results in immediate treatment as part of current practice.

For peer review only

		- Tissue injury to limb or vital organ such as perforated viscus or ischaemic limb injury.		
**Extracorporeal life support**	Extracorporeal life support following surgery and before discharge from the tertiary hospital, including the rare cases when a child was on extracorporeal life support before surgery.	This morbidity is defined by the presence of an extracorporeal life support system connected to the patient following the operation, whether it was placed in the operating theatre or in the intensive care unit, and whether the indication was cardiac arrest, low cardiac output state, poor cardiac function, arrhythmia, residual or recurrent cardiac lesion, pulmonary including pulmonary hypertension or sepsis.	It is recognised that children on extracorporeal life support following paediatric cardiac surgery have high rates of other complications including renal support, bleeding, sepsis, sternal reopening, and cardiac arrest. (4) Where such complications arise as part of extracorporeal life support, the morbidity is defined as extracorporeal life support.	The morbidity is in fact a treatment modality offered so this is not applicable. Centres offering extracorporeal life support follow protocols based on those provided by the extracorporeal life support organisation. [24]
**Necrotising enterocolitis**	Necrotising enterocolitis as a new diagnosis from after surgery until discharge from the tertiary hospital.	Necrotising enterocolitis class 1a or 1b, (5) which incorporates babies with systemic signs of inflammation and abdominal clinical signs such as distension or larger than normal gastric aspirates or mild rectal	Data in respect of systemic clinical signs, intestinal signs and radiology will be collected, as well as the	Consultation with general surgery and further management in respect of antibiotics,

For peer review only

		bleeding but no radiological changes are included, if a general surgery specialist has seen the child and commenced a course of intravenous antibiotics and parenteral nutrition for five to seven days. Cases of severe necrotising enterocolitis with radiological signs systemic instability and bowel perforation are also included.	treatments deployed, thus enabling the necrotising enterocolitis diagnosis to be graded between 1a and 3b. [25]	nutrition, radiological investigation and surgical intervention.
**Surgical site infection and bloodstream infection**	Surgical site and blood stream infections diagnosed within the hospital admission following surgery or following readmission to the same unit during post-operative recovery, where the treating clinical team assesses the infection to be linked to the recent operation. It is noted that mediastinitis may be detected more than 30 days after cardiac surgery (6) hence this time cut off is not applicable.	Deep surgical site Infection and/or mediastinitis includes any infection of an incised wound that undergoes any re intervention by a surgeon (such as opening of the wound, vacuum dressing), mediastinitis and false aneurysm, independent of culture positivity. [23]<br><br>Blood stream infection includes both catheter related and non-catheter related. Cases have systemic signs of infection, a positive culture not judged to be a contaminant, and in the case of line related a catheter in place with positive cultures from the line or from the line tip when removed.	Deep surgical site infection excludes superficial site infection managed without a surgeon's reoperation by conventional nurse dressing only, even if the wound heals by secondary intention.	The minimum treatment protocol consists of antibiotics based on organism and sensitivities, and where relevant the removal of the line. Surgical intervention may be required for deep surgical site and in some cases of endocarditis. Both conditions require

For peer review only

		Endocarditis based on clinical, imaging or culture evidence judged to be diagnostic of endothelial/endocardial infection and its sequelae cardiac or extra-cardiac.		prolonged antibiotic therapy.
**Prolonged pleural effusion or chylothorax**	Prolonged pleural effusion is a post procedural effusion with duration greater than ten days. Chylothorax is diagnosed from after surgery until discharge from the tertiary hospital.	Either a chylous pleural effusion or significant chylous pericardial effusion or significant chylous ascites or a prolonged non-chylous effusion that necessitates thoracic drainage at least ten days following index cardiac surgery.	Chylous effusions are characterised by milky appearance and a pleural fluid white blood cell count of greater than 1000 cells/µl with lymphocytes greater than 80%. (7) If the child is on normal feeds the triglyceride level in the pleural fluid will be > 1.1 mmol/L or the ratio between the pleural triglyceride level and the serum triglyceride level will exceed 1.	Diet consisting of medium chain triglycerides or low fat for chylothorax. On a patient-by-patient basis other treatments include parenteral nutrition, octreotide infusion, intervention for venous obstruction thoracic duct ligation, and pleuradhesis.

For peer review

1. Dominguez TE, Wernovsky G, Gaynor JW. Cause and prevention of central nervous system injury in neonates undergoing cardiac surgery. *Seminars in thoracic and cardiovascular surgery*. 2007;**19**:269-77 doi: 10.1053/j.semtcvs.2007.07.005 [published Online First: 2007/11/07].

2. Medoff-Cooper B, Naim M, Torowicz D, Mott A. Feeding, growth, and nutrition in children with congenitally malformed hearts. *Cardiol Young*. 2010;**20 Suppl 3**:149-53 doi: 10.1017/S1047951110001228 [published Online First: 2010/11/20].

3. NHS. Never-events. In: England N, ed. *Patient Safety*. London: NHS England 2015:Patient Safety Guideline.

4. Chaturvedi RR, Macrae D, Brown KL, *et al*. Cardiac ECMO for biventricular hearts after paediatric open heart surgery. *Heart*. 2004;**90**:545-51 Online First: 2004/04/16].

5. McElhinney DB, Hedrick HL, Bush DM, *et al*. Necrotizing enterocolitis in neonates with congenital heart disease: risk factors and outcomes. *Pediatrics*. 2000;**106**:1080-7 Online First: 2000/11/04].

6. Sohn AH, Schwartz JM, Yang KY, Jarvis WR, Guglielmo BJ, Weintrub PS. Risk factors and risk adjustment for surgical site infections in pediatric cardiothoracic surgery patients. *Am J Infect Control*. 2010;**38**:706-10 doi: 10.1016/j.ajic.2010.03.009 [published Online First: 2010/07/08].

7. Zuluaga MT. Chylothorax after surgery for congenital heart disease. *Curr Opin Pediatr*. 2012;**24**:291-4 doi: 10.1097/MOP.0b013e3283534b7f [published Online First: 2012/04/14].

For peer review only

For peer review only

STROBE Statement—checklist of items that should be included in reports of observational studies

	Item No	Recommendation
**Title and abstract**	1	(*a*) Indicate the study's design with a commonly used term in the title or the abstract
		(*b*) Provide in the abstract an informative and balanced summary of what was done and what was found
**Introduction**		
Background/rationale	2	Explain the scientific background and rationale for the investigation being reported
Objectives	3	State specific objectives, including any prespecified hypotheses
**Methods**		
Study design	4	Present key elements of study design early in the paper
Setting	5	Describe the setting, locations, and relevant dates, including periods of recruitment, exposure, follow-up, and data collection
Participants	6	(*a*) *Cohort study*—Give the eligibility criteria, and the sources and methods of selection of participants. Describe methods of follow-up<br>*Case-control study*—Give the eligibility criteria, and the sources and methods of case ascertainment and control selection. Give the rationale for the choice of cases and controls<br>*Cross-sectional study*—Give the eligibility criteria, and the sources and methods of selection of participants
		(*b*) *Cohort study*—For matched studies, give matching criteria and number of exposed and unexposed<br>*Case-control study*—For matched studies, give matching criteria and the number of controls per case
Variables	7	Clearly define all outcomes, exposures, predictors, potential confounders, and effect modifiers. Give diagnostic criteria, if applicable
Data sources/ measurement	8*	For each variable of interest, give sources of data and details of methods of assessment (measurement). Describe comparability of assessment methods if there is more than one group
Bias	9	Describe any efforts to address potential sources of bias
Study size	10	Explain how the study size was arrived at
Quantitative variables	11	Explain how quantitative variables were handled in the analyses. If applicable, describe which groupings were chosen and why
Statistical methods	12	(*a*) Describe all statistical methods, including those used to control for confounding
		(*b*) Describe any methods used to examine subgroups and interactions
		(*c*) Explain how missing data were addressed
		(*d*) *Cohort study*—If applicable, explain how loss to follow-up was addressed<br>*Case-control study*—If applicable, explain how matching of cases and controls was addressed<br>*Cross-sectional study*—If applicable, describe analytical methods taking account of sampling strategy
		(*e*) Describe any sensitivity analyses

Continued on next page

For peer review only - http://bmjopen.bmj.com/site/about/guidelines.xhtml

For peer review only

**Results**		
Participants	13*	(a) Report numbers of individuals at each stage of study—eg numbers potentially eligible, examined for eligibility, confirmed eligible, included in the study, completing follow-up, and analysed
		(b) Give reasons for non-participation at each stage
		(c) Consider use of a flow diagram
Descriptive data	14*	(a) Give characteristics of study participants (eg demographic, clinical, social) and information on exposures and potential confounders
		(b) Indicate number of participants with missing data for each variable of interest
		(c) *Cohort study*—Summarise follow-up time (eg, average and total amount)
Outcome data	15*	*Cohort study*—Report numbers of outcome events or summary measures over time
		Case-control study—Report numbers in each exposure category, or summary measures of exposure
		Cross-sectional study—Report numbers of outcome events or summary measures
Main results	16	(*a*) Give unadjusted estimates and, if applicable, confounder-adjusted estimates and their precision (eg, 95% confidence interval). Make clear which confounders were adjusted for and why they were included
		(*b*) Report category boundaries when continuous variables were categorized
		(*c*) If relevant, consider translating estimates of relative risk into absolute risk for a meaningful time period
Other analyses	17	Report other analyses done—eg analyses of subgroups and interactions, and sensitivity analyses
**Discussion**		
Key results	18	Summarise key results with reference to study objectives
Limitations	19	Discuss limitations of the study, taking into account sources of potential bias or imprecision. Discuss both direction and magnitude of any potential bias
Interpretation	20	Give a cautious overall interpretation of results considering objectives, limitations, multiplicity of analyses, results from similar studies, and other relevant evidence
Generalisability	21	Discuss the generalisability (external validity) of the study results
**Other information**		
Funding	22	Give the source of funding and the role of the funders for the present study and, if applicable, for the original study on which the present article is based

*Give information separately for cases and controls in case-control studies and, if applicable, for exposed and unexposed groups in cohort and cross-sectional studies.

Note: An Explanation and Elaboration article discusses each checklist item and gives methodological background and published examples of transparent reporting. The STROBE checklist is best used in conjunction with this article (freely available on the Web sites of PLoS Medicine at http://www.plosmedicine.org/, Annals of Internal Medicine at http://www.annals.org/, and Epidemiology at http://www.epidem.com/). Information on the STROBE Initiative is available at www.strobe-statement.org.

For peer review only

BMJ Open

What are the important morbidities associated with paediatric cardiac surgery? : A mixed methods study

Journal:	*BMJ Open*
Manuscript ID	bmjopen-2018-028533.R2
Article Type:	Research
Date Submitted by the Author:	12-Jul-2019

Complete List of Authors:	Brown, Katherine; Great Ormond Street Hospital for Children, Cardiorespiratory Division<br>Pagel, Christina; UCL<br>Ridout, Deborah; UCL, Institute of Child Health<br>Wray, Jo; Great Ormond Street Hospital for Children, Cardiorespiratory Division<br>Anderson, David; Evelina London<br>Barron, David; Birmingham Women's and Children's NHS Foundation Trust<br>Cassidy, Jane; Birmingham Women's and Children's NHS Foundation Trust<br>Davis, Peter; Bristol Royal Hospital for ChilDr.en, Paediatric Intensive Care<br>Hudson, Emma; Health Economics, University College London<br>Jones, Alison; Birmingham Women's and Children's NHS Foundation Trust<br>Mclean, Andrew; Royal Hospital for Children, Congenital Heart Surgery<br>Morris, Stephen; University College London, Department of Applied Health Research<br>Rodrigues, Warren; Royal Hospital for Children Glasgow<br>Sheehan, Karen; Bristol Royal Hospital for Children<br>Stoica, Serban ; University Hospitals Bristol NHS Foundation Trust<br>Tibby, Shane; Evelina Childrens Hospital, Paediatric Intensive Care<br>Witter, Thomas; Evelina London Children's Healthcare<br>Tsang, Victor; Great Ormond Street Hospital for Children, Cardiorespiratory Division

**Primary Subject Heading**:	Surgery
Secondary Subject Heading:	Cardiovascular medicine, Paediatrics
Keywords:	PAEDIATRICS, Cardiac surgery < SURGERY, complications, quality of life

SCHOLARONE™
Manuscripts

What are the important morbidities associated with paediatric cardiac surgery? : A mixed methods study

Authors

Katherine L Brown MD[1], Christina Pagel PHD[2], Deborah Ridout MSc[3], Jo Wray PHD[1], David Anderson FRCS[4], David J Barron MD[5], Jane Cassidy MRCP[5], Peter Davis FRCPCH[6], Emma Hudson MSc[8], Alison Jones MSc[5], Andrew McLean FRCS[10], Stephen Morris PHD[8], Warren Rodrigues MRCPCH[10], Karen Sheehan MSc[6], Serban Stoica FRCS[6], Shane Tibby MRCP[4], Thomas Witter RN[4], Victor T Tsang FRCS[1]

[1]Charles West Division, Great Ormond Street Hospital NHS Foundation Trust, Great Ormond Street, London

[2]Clinical Operational Research Unit, University College London, London

[3]Population, Policy and Practice Programme, UCL Great Ormond Street Institute of Child Health, London

[4]Department Paediatric Cardiology and Cardiac Surgery, Evelina London Children's Hospital, London

[5]Department of Intensive care and Paediatric Cardiac Surgery, Birmingham Children's Hospital, Birmingham

[6]Paediatric Intensive Care Unit and Department Paediatric Cardiac Surgery, Bristol Royal Hospital for Children, Bristol

[7]Paediatric Cardiology Department, Royal Brompton and Harefield NHS Foundation Trust, London

[8]Health Economics, Department of Applied Health Research, UCL

[9]Community Child Health, UCL Great Ormond Street Institute of Child Health, London

[10]Department of Intensive care, Royal Hospital for Children, Glasgow

Corresponding author

Katherine L Brown Great Ormond Street Hospital NHS Foundation Trust, Great Ormond Street, London WC1N 3JH

Katherine.brown@gosh.nhs.uk

Phone 0207 405 9200 extension 8016 or 8180

Fax 0207 829 8673

For peer review only - http://bmjopen.bmj.com/site/about/guidelines.xhtml

Keywords – Paediatrics, cardiac surgery, outcome, complications, morbidity, quality of life

Word count 3575

For peer review only

For peer review only - http://bmjopen.bmj.com/site/about/guidelines.xhtml

Abstract

Objectives:

Given the current excellent early mortality rates for paediatric cardiac surgery, stakeholders believe that this important safety outcome should be supplemented by a wider range of measures. Our objectives were to prospectively measure the incidence of morbidities following paediatric cardiac surgery and to evaluate their clinical and health-economic impact over 6-months.

Design:

The design was a prospective, multi-centre, multidisciplinary mixed methods study.

Setting:

The setting was five of the ten paediatric cardiac surgery centres in the United Kingdom with 21 months recruitment.

Participants:

Included were 3090 paediatric cardiac surgeries, of which 666 patients were recruited to an impact sub study.

Results:

Families and clinicians prioritized:

Acute neurological event, unplanned re-intervention, feeding problems, renal replacement therapy, major adverse events, extracorporeal life support, necrotising enterocolitis, post-surgical infection, and prolonged pleural effusion or chylothorax.

Amongst 3090 consecutive surgeries there were 675 (21.8%) with at least one of these morbidities. Independent risk factors for morbidity included neonatal age, complex heart disease and prolonged cardiopulmonary bypass (P<0·001). Amongst patients with morbidity, 6-month survival was 88·2% (95% CI 85·4, 90·6) compared to 99·3% (95% CI 98·9, 99·6) with none of the morbidities (P<0·001).The impact sub-study in 340 children with morbidity and 326 control children with no morbidity, indicated that morbidity related impairment in quality of life improved between 6 weeks and 6 months. When compared to children with no morbidities, those with morbidity experienced a median of 13 (95% CI (10.2, 15.8) P<0·001) fewer days at home by 6 months, and an adjusted incremental cost of £21,292, (95% CI (£17,694, £32,423) P<0.001).

For peer review only - http://bmjopen.bmj.com/site/about/guidelines.xhtml

For peer review only

Conclusions:

Evaluation of post-operative morbidity is more complicated than measuring early mortality. However, tracking morbidity after paediatric cardiac surgery over 6 months offers stakeholders important data that is of value to parents and will be useful in driving future quality improvement.

Strengths and limitations of this study

- Our study is unique, given that the morbidity measures selected for study reflect the viewpoints of both lay people and clinicians, whereas previous work has tended to focus on clinical metrics only.
- A limitation of our study is that although we were able to identify broad risk factors for morbidity after paediatric cardiac surgery, a larger dataset will be needed to generate more reliable risk adjustment methods.
- A strength of our study was that it was multi-centre, prospective, and followed children up for 6 months after their operation, which is a longer time horizon than the usual 30-day period.

For peer review only

For peer review only - http://bmjopen.bmj.com/site/about/guidelines.xhtml

Study registration

The study has ethical approval from London City Road Research Ethics Committee (14-LO-1442).

Funding details

This project was funded by the National Institute for Health Research Health Services and Delivery Research programme (Project No: 12/5005/06). The views and opinions expressed therein are those of the authors and do not necessarily reflect those of the NIHR HS&DR programme or the Department of Health. K Brown, D Ridout, J Wray, and V Tsang were supported by the National Institute for Health Research Biomedical Research Centre at Great Ormond Street Hospital for Children NHS Foundation Trust and University College London. The funder had no role in the study.

Conflicts of interest

None declared by authors.

Data sharing

For data to be made available specific ethical and HRA approval would be required.

For peer review only

Background

Over 5000 paediatric cardiac surgery procedures are performed in the UK each year and early survival has improved to over 98% since comprehensive national audit commenced in 2000. [1 2] Most stakeholders including clinicians, commissioners and users believe that whilst these early survival rates remain an important safety measure, it is has become imperative to explore a broader range of measures for outcome in this complex field of practice. Whilst there has been considerable research related to measuring, understanding and reducing peri-operative mortality for paediatric cardiac surgery, [3-5]much less attention has been focussed on surgical morbidities.

Morbidity is defined as a state of being unhealthy, or of experiencing an aspect of health that is 'generally bad for you'. In this project, by 'morbidity' we mean a defined aspect of ill health associated with a specific operation. In our study, we aimed to identify which morbidities present the greatest burden on patients and health services following paediatric cardiac surgery. Views may differ between professionals and non-professionals as to what the term morbidity exactly refers to and which surgical morbidities are most important. [6] Therefore we set out to combine patient and carers' perspectives with those of professional groups in defining a prioritised list of morbidities for prospective evaluation.

Over four years we:

1. Identified and defined nine morbidities following paediatric heart surgery, taking into account views from patients, carers, psychologists, nurses and clinicians, which together captured important aspects of the clinical and health-economic burden;
2. Measured incidence of the defined morbidities in the UK patient population and in subgroups defined by case complexity;
3. Evaluated the impact of defined morbidities on quality of life and estimated their clinical and health economic burden.

Methods

Design

The design was a prospective, multi-centre, multidisciplinary mixed methods study. Selection of morbidities was based on consensus methods. Morbidity incidence was evaluated with a prospective cross sectional study. Measurement of morbidity impact entailed a prospective, case-matched, longitudinal study.

For peer review only - http://bmjopen.bmj.com/site/about/guidelines.xhtml

Setting

Five UK paediatric cardiac surgery centres representing a range of programme sizes, which care for half of all patients nationally.

Participants

The participants were children under 17 years old with congenital heart disease (CHD).

Consent

Parents and children participating in focus groups and in the prospective study of morbidity impact provided written informed consent.

Which morbidities?

Between January 2014 and September 2015, we reviewed existing literature, ran three family focus groups and undertook a family online discussion forum moderated by the Children's Heart Federation (a user group). Transcripts were thematically analysed and the resultant themes, together with the literature, helped to inform a long list of candidate surgical morbidities. A multi-disciplinary group, with patient and carer representation, then ranked and selected a list of nine key morbidities using the nominal group technique and secret voting.[7] This 'selection panel' [7] was informed in turn by clinical views on definitions and feasibility of routine monitoring for each candidate morbidity provided by an independent 'definition panel' [8] as reported in previous publications.

Incidence of morbidity

Between October 2015 and June 2017, we prospectively measured instances of morbidity within all consecutive surgical admissions across the study sites. Appendix 1 [8] provides the details of the criteria followed to define each individual morbidity, including the timeframe for diagnosis. Morbidities were attributed to the immediately preceding cardiac surgery and defined within the same hospitalisation. The only exceptions were unplanned re operation, which was defined as an unanticipated procedure within 30-days and mediastinitis, which could be identified post-discharge by the operating surgeon. [8] Data were regularly checked for completeness, clinical congruence and accuracy and, a three month sample of data was independently validated against the national audit data.

As for the UK audit of 30-day mortality, [9] all procedures more than 30 days apart on the same patient, were included in the morbidity analysis. As a secondary outcome, we checked each patient's survival status at 6 months after first appearance in the dataset, based on the

For peer review only - http://bmjopen.bmj.com/site/about/guidelines.xhtml

individual centres National Congenital Heart Diseases Audit (NCHDA) [2] data and local hospital records.

Impact of morbidity

Recruitment

Recruitment to the impact study ran between October 2015 and June 2017 for all but one site, where it stopped after 6 months due to resource constraints. We attempted to recruit all patients with morbidity from the wider population, when at least one parent spoke reasonable English, and the family were resident in the UK. When feasible, for each morbidity case, we recruited a patient with no morbidity, matched on treating centre, age and univentricular status. The recruitment strategy led to the recruitment of a sample that was evenly balanced between patients with morbidity and those with no morbidity, however we were not able to find a match for every morbidity-patient. In order to account for the widest possible spectrum of important outcomes we included the available data on children who subsequently died.

Measures of impact

We evaluated the impact of morbidities over the 6 months following surgery, based on the following outcomes:

a) Quality of life and psychological burden on children and parents

We used age-specific measures at 6 weeks and 6 months following surgery. These were the PedsQL subscales (physical and psychosocial) and total scores. These scores range from 0 and 100, a higher score indicating better quality of life. PedsQL scores in a normal healthy population vary by age; expected scores for infants 0-12 months (which encompasses the median age for our cohort) are mean physical 84·98 (SD 9·45), mean psychosocial 80·47 (SD 12·64) and mean total 82·47 (SD 9·95). [10-12]

For the parents, we used the PHQ-4, which comprises four questions, measuring anxiety and depression in adults. Individual items are scored from zero to three. Scores ≥three for the first two questions suggest anxiety, and scores ≥three for last two questions suggest depression. [13] In the normal population, 4·8% have scores suggestive of anxiety and 6·6% have PHQ-4 scores suggestive of depression. [14]

b) Days at home over 6 months as an additional measure of disruption to family life

Length of inpatient stay following the index procedure was defined as the number of days between the operation and the date of discharge from the tertiary centre. Hospital stays that exceeded the 6-month follow-up period were censored after 183 days. All subsequent

For peer review only - http://bmjopen.bmj.com/site/about/guidelines.xhtml

hospital admissions were captured and the total days spent in hospital including tertiary and secondary care admissions was subtracted from 183 days to generate a value for days at home by 6 months. Children who died in hospital were assigned a value of zero for this outcome.

c) Costs of the index hospitalisation

We calculated the cost of the inpatient stay in the tertiary centre following the index surgical procedure as a key indicator of the economic burden to the hospital provider (measured in 2016/17 UK£).

Costs were calculated as the summation of days in intensive care unit (ICU), days on the ward, and all operating procedures within the defined period. For ICU stays, data were recorded on the level of care the patient received each day (Enhanced Care, High Dependency, High Dependency Advanced, ICU Basic, ICU Basic Enhanced, ICU Advanced, ICU Advanced Enhanced and ECLS). [15] Unit costs of ICU stays were daily costs for each level of care (ranging from £870 to £5440) applied to the number of days spent receiving that level of care. Unit costs per day for ward stays were obtained from the highest recruiting centre and varied according to the age of the patient (<two years, £904; ≥two years, £680). Unit costs for surgical procedures were costs per minute supplied by study sites; these varied by site and so we used the value for the site that recruited the largest number of patients to this study (£13·39 per minute), which was similar to the mean value across all sites.

Statistical and Health Economic Analyses

Patient sample size

We anticipated that between 3000 and 3300 surgical patients would be admitted across the five sites during the study period. [2] We assumed that a clinically relevant difference in quality of life between matched pairs corresponded to a mean difference in quality of life score of at least 0.5 standard deviations. [16] To detect such a difference for PedsQL responses at 5% significance with 80% power requires a minimum of 32 matched pairs. Allowing for a 10% loss to follow-up rate, we aimed to recruit 36 matched pairs for each patient with morbidity, giving 80% power to detect a significant effect for any morbidity with a prevalence of at least 1·5%.

Analysis of risk factors for morbidity

Based on the whole sample of cardiac surgeries across the five sites over the study period, we explored risk factors for occurrence of morbidity. Clinical risk factor groups were derived from the finer diagnostic coding, based on previous peer reviewed research by our group. [9 17]

For peer review only - http://bmjopen.bmj.com/site/about/guidelines.xhtml

We used multilevel logistic regression analysis to explore the role of pre-operative, patient-level case mix factors on the occurrence of any morbidity versus no morbidity, accounting for multiple procedures within patients. Firstly a univariate model to predict risk of any morbidity vs none was fitted for each risk factor. The estimated Odds ratios (OR) are presented along with 95% confidence intervals (CI). Then all factors significant on univariate analysis ($p<0 \cdot 1$) were considered in a multivariable model. We used multiple imputation by chained equations to account for missing data. The imputation model included all risk factors considered in the univariate analysis, which we assumed included all predictors of missingness. We indicate missing data in our results. The multivariable models were derived by fitting a regression model for all significant predictors and estimates were combined using Rubin's rules. [18]

Secondary outcome

The secondary outcome of survival at 6 months was calculated for all patients who had cardiac surgery at the sites within the study period. Unadjusted 6-month mortality rates were compared between morbidity groups using logistic regression.

Analysis of the impact of morbidity

In the patients recruited to the impact study, we analysed the 'impact outcomes' over 6 months post-surgery.

We used mixed effects regression models for PedsQL/PHQ-4 results to compare the impact of morbidity (any versus none) on outcome. All models were adjusted for clustering within matched pairs and significant covariates associated with incidence of morbidity. We used multiple imputation, by chained equations to account for missing data for those patients known to be alive at 6 weeks and 6 months respectively.

We used quantile regression to estimate the effects of individual morbidities in terms of differences in median days at home by 6 months, since the measure was negatively skewed. All models were adjusted for clustering within matched pairs and significant covariates associated with incidence of morbidity. Given the high level of data completeness, imputation was not used.

The inclusion of hospital site made negligible difference to either of these analyses, and not included in the statistical models.

For peer review only - http://bmjopen.bmj.com/site/about/guidelines.xhtml

We modelled the relationship between morbidities and cost of index hospitalisation, adjusted for significant covariates associated with hospital costs. Costs were skewed and to account for this we used a generalised linear model (GLM) with gamma family and log link, which has been recommended for modelling positively skewed data. [19] Missing outcome values were imputed using an iterative Markov chain Monte Carlo procedure based on multivariate normal regression, generating 20 imputed datasets. The hospital site was included as a significant covariate in the health economic model.

All analyses were performed in Stata 14. [20]

Patient and public involvement

The patient or user perspective lies at the core of the study methodology. Specifically, a key goal of the study was to consider the views of patients and parents when measures of morbidity are selected for future audit and benchmarking, in particular since emphasis may potentially differ between professionals and parents / patients.

Patient and family representatives from the Children's Heart Federation (CHF) were involved in aspects of the study design including design and running of the focus groups and the online discussion forum.

Representatives of the CHF participated in the facilitated nominal group meetings to select morbidities for inclusion in the incidence and impact studies.

A member of the CHF and a parent representative sat on the project steering group.

Results

1: Which morbidities?

Our final selected [7] and defined [8] list of included morbidities were:

Acute neurological event, unplanned re-intervention, feeding problems, renal replacement therapy, major adverse events, extracorporeal life support (ECLS), necrotising enterocolitis, post-surgical infection and prolonged pleural effusion or chylothorax.

We treated children with more than one of these events as a distinct group referred to as 'multi-morbidity'. Recognising ECLS as a very severe event, which nearly always occurs with other morbidities, we treated ECLS as standalone morbidity (and always excluded from the multiple morbidity group). [21-25]

For peer review only - http://bmjopen.bmj.com/site/about/guidelines.xhtml

For peer review only

There was some divergence between the views of clinicians and families about the fundamental issue of what the important morbidities linked to paediatric cardiac surgery are. Health professionals from tertiary centres tended to prioritise clearly clinical issues related to the heart (e.g. use of ECLS and re operation) whereas parents placed greater emphasis on holistic outcomes for their child (e.g. feeding difficulties and child developmental problems). [7]

2. Incidence of morbidity

Descriptive results

We collected data on 3248 cardiac procedures and analysed 3090, after excluding 99 cardiac re-operations (a morbidity outcome) and 59 procedures that did not meet inclusion criteria.

The incidence of individual morbidities in isolation and overall for each is shown in Figure 1.

Figure 1 Incidence of morbidities

The figure shows the distinction between occurrence as a single morbidity and occurrence in combination with other morbidities, with 95% confidence intervals.

For peer review only

Significant risk factors for morbidity

As shown in Table 1, the most important risk factor for occurrence of 'any morbidity' was young age, particularly neonates, who were approximately five times as likely to experience a morbidity compared to children over one year old. Children with more complex heart disease or children whose operation included a bypass time longer than 90 minutes were almost twice as likely to experience morbidity. Other important independent risk factors were: palliative or staged procedures, the presence of functionally univentricular heart and pre-procedure severity of illness factors (e.g. pre-procedure mechanical ventilation or shock).

Table 1Summary of risk factors by any morbidity outcome

The weight was missing in 87 (2·8%) procedures and weight for age was set to missing for an additional 99 (3·2%) patients in whom the value was >5 SD from the normative mean. Other data were complete. Values include imputed data.

Cardiac diagnosis group A) hypoplastic left heart syndrome, Truncus arteriosus, pulmonary atresia intact septum, B) Functionally univentricular heart, pulmonary atresia ventricular septal defect, C) transposition of the great arteries all types, interrupted aortic arch, totally anomalous pulmonary venous connection, D) patent ductus arteriosus, tricuspid valve anomalies, acquired heart disease, complete atrioventricular septal defect, E) Tetralogy of Fallot, mitral valve anomalies, isolated aortic stenosis, aortic regurgitation, aortic arch obstruction, subaortic obstruction, ventricular septal defect, atrial septal defect.

The results of the multiple logistic regression model for occurrence of 'any morbidity', are expressed as odds ratio for 'any morbidity' by the stated factor, adjusted for age band, low weight, cardiac diagnostic category, functionally univentricular heart, acquired comorbidity, congenital comorbidity, severity of illness indicators, additional cardiac risk factors, specific procedure group, bypass time category.

	None of the morbidities (N = 2415)	Any Morbidity (N=675)	Univariate odds ratio for 'any morbidity' (95% CI), P Value	Multivariable odds ratio for 'any morbidity' (95% CI), P Value
**Male**	1299 (53.8)	372 (55.1)	1.05 (0.89, 1.25), P=0.54	-
**Median Age (days) (IQR)**	286 (105, 1582)	102 (10, 331)		
**Child**	1111 (46.0)	160 (23.7)		
**Infant**	1023 (42.4)	268 (39.7)	1.82 (1.47, 2.25), P<0.001	1.61 (1.26, 2.05), P<0.001
**Neonate**	281 (11.6)	247 (36.6)	6.10 (4.81, 7.75)< P<0.001	5.26 (3.90, 7.09), P<0.001
**Median Weight (Kg) (IQR)**	7.7 (4.7, 16.2)	4.6 (3.2, 8.0)		
**Low weight <2 SD below mean**	714 (31.5)	234 (36.8)	1.27 (1.05, 1.52)· P=0.01	1.21 (0.97, 1.51), P=0.09
**Cardiac Diagnosis Group**				
**E Least complex (reference)**	1002 (41.5)	123 (18.2)		
**D**	796 (33.0)	227 (33.6)	5.13 (3.79, 6.93), P<0.001	2.02 (1.58, 2.60), P<0.001
**C**	215 (8.9)	109 (16.2)	3.83 (2.85, 5.13), P<0.001	1.44 (1.00, 2.07), P=0.05
**B**	232 (9.6)	109 (16.2)	4.13 (3.07, 5.55), P<0.001	2.62 (1.85, 3.71), P<0.001
**A Most Complex**	170 (7.0)	107 (15.8)	2.32 (1.83, 2.94), P<0.001	2.14 (1.41, 3.24), P<0.001
**Functionally univentricular heart**	255 (10.6)	159 (23.6)	2.61 (2.11, 3.23), P<0.001	1.55 (1.07, 2.24), P=0.02
**Acquired comorbidity**	337 (14.0)	119 (17.6)	1.32 (1.05, 1.66), P=0.02	1.33 (1.03, 1.71), P=0.03
**Congenital comorbidity**	537 (22.2)	178 (26.4)	1.25 (1.03, 1.52), P=0.03	1.28 (1.02, 1.59), P=0.03
**Severity of illness indicators**	222 (9.2)	152 (22.5)	2.87 (2.30, 3.58), P<0.001	1.52 (1.16, 2.00), P<0.01
**Premature <37 weeks gestation**	231 (9.6)	73 (10.8)	1.15 (0.87, 1.51), P=0.33	-
**Downs Syndrome**	214 (8.9)	63 (9.3)	1.06 (0.79, 1.43), P=0.71	-
**Additional cardiac risk**	165 (6.8)	65 (9.6)	1.45 (1.09, 1.94), P=0.01	1.39 (0.99, 1.94), P=0.05
**Surgical procedure type**				
**Reparative/corrective (reference)**	1391 (57.6)	332 (49.2)		
**Palliative/staged**	331 (13.7)	179 (26.5)	2.27 (1.82, 2.82), P<0.001	1.65 (1.14, 2.38), P<0.01
**Ungrouped**	693 (28.7)	164 (24.3)	0.99 (0.81, 1.22), P=0.94	1.04 (0.82, 1.31), P=0.75
**Median bypass time (mins) (IQR)**	72 (42, 110)	110 (62, 156)		
**No bypass (reference)**	390 (16.2)	103 (15.3)		
**Up to 90 mins**	1148 (47.5)	150 (22.2)	0.48 (0.35, 0.65), P<0.001	0.78 (0.57, 1.09), P=0.14
**More than 90 mins**	877 (36.3)	422 (62.5)	1.76 (1.32, 2.34), P<0.001	2.28 (1.67, 3.12), P<0.001

For peer review only - http://bmjopen.bmj.com/site/about/guidelines.xhtml

Secondary outcomes

Patient survival was highest and patient hospital stay was shortest for patients with none of the morbidities (see Table 2). The 6-month survival was significantly lower for patients with a single morbidity, for those with ECLS and multiple morbidities, compared with patients with none of the morbidities P < 0·001 for all.

Table 2 Secondary outcomes of hospital stay and survival at 6 months by morbidity

The number (%) of morbidities was based on individual surgical episode, as was the length of stay, whereas survival was calculated per patient. Data are presented based on morbidity in isolation, multi-morbidity and ECLS. Length of stay data was missing for 9 patients and Life status was missing in 16 patients who were not included.

Morbidity type	Number (%)	Median length of post-operative hospital stay in days (IQR)	6 month survival Number, %, (95% CI)
**No morbidity**	2415 (78.2%)	8 days (IQR 5, 13)	2202/2217 99.3% (98.9, 99.6)
**Any morbidity**	675 (21.8%)	24 days	675 (21.8%)
**Acute neurological event**	14 (0.5)	19 days (12, 39)	12/13 92.3% (64.0, 99.8)
**Unplanned re-intervention**	59 (1.9%)	22 days (14, 33)	50/54 92.6% (82.1, 97.9)
**Feeding problems**	99 (3.2%)	20.5 days (12, 36)	90/91 98.9% (94, 100)
**Renal support**	40 (1.3%)	17 days (14, 26)	37/39 94.9% (82.7, 99.4)
**Major adverse event**	34 (1.1)	16.5 days (8, 25)	28/33 84.9% (68.1, 94.9)
**Necrotising enterocolitis**	32 (1.0%)	24.5 days (18.5, 49.5)	28/30 93.3% (77.9, 99.2)
**Post-surgical infection**	27 (0.9%)	20.5 days (11, 28)	25/25 100% (86.3, 100)
**Prolonged pleural effusion**	111 (3.6%)	20 days (14, 28)	95/96 99.0% (94.3, 100)
**Multi- morbidity**	197 (6.4)	35 days (IQR 22, 56)	158/190 83.2% (77.1, 88.2)
**Extracorporeal life support**	62 (2.0%)	43 days (IQR 20, 84)	31/57 54.4% (40.7, 67.6)

3. Impact of morbidity

Patient sample

We recruited 60% of eligible families, amounting to 666 children of whom 340 (51%) had at least one morbidity. Of these, 19 died within 30 days and 39 died within 6 months. We pair matched 558 patients and 108 patients unmatched of whom 61 had morbidity; all of the patients were included in the statistical analysis.

Quality of life and anxiety - depression outcomes

For the 6-weeks data, a comparison between patients with missing versus non missing data indicated the only difference was in the proportions with severity of illness factors and

For peer review only - http://bmjopen.bmj.com/site/about/guidelines.xhtml

additional cardiac risk factors. Importantly there was no difference in the proportion of patients with or without morbidity. At the 6 months mark there was no difference in any baseline factor or morbidity occurrence.

All PedQL scores were lower than in a healthy population (see methods [10-12] and Table 3). In terms of our study objective to assess the impact of morbidity; the total PedsQL scores were significantly reduced for children with any morbidity at 6 weeks in comparison to children with none of the morbidities (case mix adjusted score reduction of -5·2 (95% CI -8·3, -2·2) P<0·01). On a positive note, this difference narrowed to small non-significant reduction in total score by 6 months. Physical scores were affected more than psychosocial scores, and were still significantly impaired at 6-months.

All parents experienced higher rates of both anxiety and depression than a healthy population (see methods and Table 3). The parents of children with morbidity were around 57% more likely to experience anxiety; and around 77% more likely to experience depression, at 6 weeks post operation than parents of children without a morbidity. PHQ-4 scores had improved by 6 months, and although there remained a higher chance of both anxiety and depression with morbidity, the difference narrowed and was not significant.

For peer review only - http://bmjopen.bmj.com/site/about/guidelines.xhtml

Table 3: Quality of life and parental anxiety / depression based on whether or not a morbidity was present

Quality of life (QOL) was derived from PedsQL scores for 478 children at 6 weeks (26% missing) and 403 children at 6 months (36% missing).

Parental anxiety and depression was derived from PHQ-4 responses of 481 parents at 6 weeks (26% missing) and 394 parents at 6 months (37% missing).

Values include imputed data.

The differences in each outcome are adjusted for age band, low weight, cardiac diagnostic category, functionally univentricular heart, acquired comorbidity, congenital comorbidity, severity of illness indicators, additional cardiac risk factors, specific procedure group, bypass time category.

The impact outcome type and time point of measurement	Patients with none of the morbidities	Patients with any morbidity	Differences in outcome based on presence of any morbidity
	Score Mean (SD)	*Score Mean (SD)*	*Adjusted difference in mean score with morbidity (95% CI), P value*
**Physical QOL Score at 6 weeks**	79.0 (16.2)	69.1 (21.8)	-8.3 (-11.8, -4.9), P<0.001
**Psychosocial QOL Score at 6 weeks**	79.4 (14.7)	75.2 (19.0)	-2.7 (-5.9, 0.5), P=0.08
**Total QOL Score at 6 weeks**	79.3 (13.8)	72.1 (19.1)	-5.2 (-8.3, -2.2), P<0.01
	Score Mean (SD)	*Score Mean (SD)*	*Adjusted difference in mean score with morbidity* (95% CI), P value
**Physical QOL Score at 6 months**	82.3 (16.6)	76.6 (18.6)	-4.2 (-7.6, -0.8), P=0.02
**Psychosocial QOL Score at 6 months**	78.0 (14.7)	76.4 (15.4)	-0.9 (-3.5, 1.8), P=0.5
**Total QOL Score at 6 months**	79.8 (13.9)	76.6 (15.0)	-2.3 (-4.9, 0.3), P=0.08
	Number (%) with attribute	*Number (%) with attribute*	*Adjusted odds ratio for outcome by morbidity* (95% CI)
**Parental Anxiety at 6 weeks**	55 (23.7)	92 (36.8)	1.57 (1.04, 2.35), P=0.03
**Parental Depression at 6 weeks**	26 (11.2)	55 (22.1)	1.77 (1.10, 2.86), P=0.02
	Number (%) with attribute	*Number (%) with attribute*	*Adjusted odds ratio for outcome by morbidity (95% CI)*
**Parental Anxiety at 6 months**	23 (11.5)	34 (17.4)	1.37 (0.77, 2.43), P=0.28
**Parental Depression at 6 months**	15 (7.5)	23 (11.9)	1.45 (0.79, 2.68), P=0.23

Days at home by 6 months outcome

Children with none of the morbidities had a median of 174 days at home (IQR 169, 176) out of a possible 183 days in 6 months. The presence of any morbidity reduced the median days at home by 13 days over 6 months (95% CI (10·2, 15·8) P<0·001). When individual morbidities were each considered in turn, the difference in the case mix adjusted median days at home was significantly lower at 6 months for all of them apart from 'renal support' (P<0.05).

Health economic outcome

The costs of the index hospitalisation were available for 613 patients (8% missing). The mean additional cost linked to having any of the selected morbidities was £21,292, (95% CI £17,694, £32,423). P<0.001. In Table 4, we present the adjusted marginal effects, which are

For peer review only - http://bmjopen.bmj.com/site/about/guidelines.xhtml

the adjusted incremental costs linked to the stated morbidity, in comparison to the adjusted cost linked to patients with none of the selected morbidities. The greatest adjusted cost difference was for ECLS, where the adjusted mean difference in costs was £62,452; followed by multi-morbidity, where the adjusted mean difference was £33,147 (P<0·01 for both). Significantly increased (P<0·05) costs were also found for unplanned re-intervention, feeding problems, renal support and prolonged pleural effusion. The remaining morbidities, representing the lowest number of patients per category, showed non-significant differences.

Table 4 Cost of hospital stay following index surgical procedure by morbidity

Figures in the Marginal Effect column are the mean difference in costs between each morbidity category and the category of no selected morbidities present, conditional on the covariates. Costs are in 2016/17 UK£.

Values include imputed data.

The marginal effects are adjusted for age band; weight-by-age z score; congenital morbidity, severity of illness indicator, Downs' syndrome; cardiac diagnosis category; cardiac procedure category; whether or not the patient died within 6 months; and, study site.

Morbidity	Coef.	Marginal Effect (£) (95% CI), P Value
**Any morbidity (n=340)**	0.71	21,292, (95% CI 17,694, 32,423). P<0.001.
**Acute neurological event (n=6)**	0.50	13,911 (-3,923, 50,059), P=0.16
**Unplanned re-intervention (n=26)**	0.62	18,330 (7,475, 33,282), P<0.01
**Feeding morbidity (n=45)**	0.55	15,541 (7,200, 26,326), P<0.01
**Renal support (n=24)**	0.41	10,798 (1,846, 23,215), P=0.01
**Major adverse event (n=22)**	0.30	7,555 (-1,131, 19,984), P=0.10
**Necrotising enterocolitis (n=11)**	0.37	9,547 (-2,173, 28,454), P=0.13
**Post-surgical infection (n=11)**	0.27	6,466 (-3,947, 23,130), P=0.17
**Prolonged pleural effusion (n=50)**	0.33	8,177 (1,444, 16,904), P=0.01
**Extracorporeal life support (n=27)**	1.37	62,452 (39,546, 93,983), P<0.01
**Multi-morbidity (n=118)**	0.94	33,147 (23,669, 44,624), P<0.01

Discussion

Overview of findings

This paper presents the results of the first prospective multi-centre study of morbidity after paediatric cardiac surgery morbidity that measured outcome 6-months after operation. The results are novel and indicate that evaluation of morbidity adds considerably to the current main metric of 30-day or in hospital survival.

For peer review only - http://bmjopen.bmj.com/site/about/guidelines.xhtml

We note the excellent outcomes in children who had none of the morbidities: their survival at 6-months post-operation was 99·3% and their median length of stay was 8 days, suggesting that the morbidities we selected do capture most of the complication-related adverse outcomes for this context.

Based on our impact sub study, occurrence of ECLS and multiple morbidities are particularly important adverse outcomes – they are associated with high mortality, high resource use and lower quality of life for surviving patients over 6 months after surgery. Future routine monitoring and public reporting for these should be considered by centres and the national audit.

In our engagement work, patients' families consistently told us that outcomes affecting the child as a whole over the medium or longer term are very important to them. We thus included morbidities such as feeding problems and acute neurological events but note that those types of outcome were the most complicated to capture, and we intend to publish additional material on these topics.

Limitations

We acknowledge that the definition, measurement, reporting and interpretation of morbidity, is much more complex than for mortality. This activity requires significant staff resources and clinician buy in, in particular because some morbidities are challenging to measure and to interpret. The number of individual morbidities was low in some of our analyses, limiting interpretation. We have not captured every single morbidity that exists after children's heart surgery.

Context

There have been previous attempts at reporting morbidity after paediatric cardiac surgery, however these have in general captured events within a hospital setting, whereas our study captures outcomes out to 6 months post-surgery. Two single-centre studies generated an aggregate 'Morbidity Index' by assigning subjective weights to post-operative complications. [26 27] The Society of Thoracic Surgery in the USA, developed a 'Morbidity Score', based on data from their multi-centre registry. [28] We note that condensing diverse morbidities into a single score may lead to loss of information. Moreover, recent work on using graphical methods to routinely monitor a range of morbidities [29] highlighted the complexity of graphically summarising multiple morbidities. [30] The Pediatric Cardiac Critical Care Consortium (PC^4) was set up in 2009, and provides partner sites with access to real-time,

For peer review only - http://bmjopen.bmj.com/site/about/guidelines.xhtml

reliable and actionable data to be used for local quality improvements. [31-34] However membership of PC[4] is voluntary for institutions on a subscription basis, the reported measures been selected by clinicians, the reporting of outcomes is accessible only to subscribing member institutions and these are limited to in hospital measures.

Future practice and research

Neurodevelopmental problems are common in children with CHD undergoing surgery [35] however we detected acute neurological events (ANE) following only 2·1% of procedures. : Therefore it appears that peri-operative 'ANE' represents the tip of the iceberg, and hence the scope of surveillance for child neurodevelopment in CHD needs to extend well beyond the peri-operative period.

Despite their great importance to families, we note that participants reported subjectivity in the collection of data on 'feeding problems'; hence these were difficult to capture consistently. Additional research may help determine the best way to measure this important morbidity and to elucidate approaches to alleviate the impact of feeding problems in CHD.

Our measure of renal failure was the need for renal support, and although relatively easy data to collect this may not be the optimal method to capture this morbidity, given that there are differences in practice between clinicians. [36] Further research may help us to understand the best approach to manage post-operative renal injury in CHD.

Our results perhaps unsurprisingly indicate that parents of children who suffer morbidities experience higher rates of anxiety and depression post-operatively. This emphasises the importance of supporting parents during this phase.

We used study data to co-develop parent and carer information resources, showing what the morbidities are and how their incidence and the length of stay may vary based on the complexity of the child's condition. Parents told us that it helps to know that; firstly they are not alone in facing a morbidity, secondly clinical teams have seen morbidities before and know how to deal with them, and thirdly that it is better as a parent to 'be prepared'. Furthermore, parents indicated that information about impact such as nearly all children who experience a morbidity and recover will have a similar quality of life to children who did not experience a morbidity by the six month mark, was very helpful to know.

Within the scope of this project, a new Excel tool has been developed and piloted, which enables clinical teams to benchmark and report the local rates of morbidities with a quality

For peer review only - http://bmjopen.bmj.com/site/about/guidelines.xhtml

assurance goal, such as in a mortality and morbidity conference. Furthermore, we have kept in close contact with the National Congenital Heart Diseases Audit (NCHDA) and the Clinical Reference Group for CHD services. NCHDA has already started to collect five of the nine morbidities within the nationally mandated dataset, using the definitions that we developed.

For peer review only

For peer review only - http://bmjopen.bmj.com/site/about/guidelines.xhtml

Author contributions

Designed the study and wrote the protocol: Katherine Brown, Christina Pagel, Deborah Ridout, Jo Wray, Stephen Morris, and Victor Tsang.

Clinical expertise: Victor Tsang, David Anderson, David Barron, Serban Stoica, Andrew Maclean, Katherine Brown, Shane Tibby, David Barron, Peter Davis and Warren Rodrigues.

Study implementation, protocol adjustments, data cleaning: Katherine Brown, Alison Jones, Karen Sheehan, Victor Tsang, Jane Cassidy, David Barron, Peter Davis, Serban Stoica, Warren Rodrigues, and Shane Tibby.

Statistical analysis: Deborah Ridout, Christina Pagel, Shane Tibby, and Katherine Brown.

Health economic analysis: Emma Hudson, Stephen Morris.

Contributed to the write up and signed off the paper: Katherine Brown, Christina Pagel, Deborah Ridout, Jo Wray, Victor Tsang, David Anderson, David Barron, Jane Cassidy, Peter Davis, Emma Hudson, Alison Jones, Andrew McLean, Stephen Morris, Warren Rodrigues, Karen Sheehan, Serban Stoica, Shane Tibby, and Thomas Witter.

Acknowledgements (includes patient and public contributor names)

We would like to thank Martin Utley, Sheryl Snowball, Luke Maidment, Sarah Bohannon, Liz Smith, Kate Penny-Thomas, Joanne Webb, Sinead Cummins, John Stickley, Natasha Khan, Teresa Dickson, Ray Samson, Isobel Mcleod, Paul Wellman, Rhian Lakhani, Kathleen Selway, Carrie Cherrington, Andrew Parry, Rob Tulloh, Bill Gaynor, Rodney Franklin, Lisa Allera, Kate Bull, Trevor Ritchens, Branko Mimic, Jon Smith, Lyvonne Tume, Vibeke Hjortdal, Michael Vath, Tom Treasure, Anne Keatley Clarke, Bea Tuten, and all the patients who participated in the study.

For peer review only - http://bmjopen.bmj.com/site/about/guidelines.xhtml

References

1. Brown KL, Crowe S, Franklin R, et al. Trends in 30-day mortality rate and case mix for paediatric cardiac surgery in the UK between 2000 and 2010. *Open Heart* 2015;2(1):e000157. doi: 10.1136/openhrt-2014-000157 [published Online First: 2015/04/22]
2. NICOR. NICOR: National Institute for Cardiovascular Outcomes Research: Congenital Heart Diseases Website UCL London UK: University College London; 2017 [Available from: www.nicor4.nicor.org.uk/CHD accessed Jan 2017 2017.
3. O'Brien SM, Clarke DR, Jacobs JP, et al. An empirically based tool for analyzing mortality associated with congenital heart surgery. *J Thorac Cardiovasc Surg* 2009;138(5):1139-53. doi: S0022-5223(09)01017-4 [pii]

10.1016/j.jtcvs.2009.03.071 [published Online First: 2009/10/20]
4. Jenkins KJ, Gauvreau K, Newburger JW, et al. Consensus-based method for risk adjustment for surgery for congenital heart disease. *J Thorac Cardiovasc Surg* 2002;123(1):110-8. doi: S0022522302909451 [pii] [published Online First: 2002/01/10]
5. Lacour-Gayet F, Clarke D, Jacobs J, et al. The Aristotle score for congenital heart surgery. *Semin Thorac Cardiovasc Surg Pediatr Card Surg Annu* 2004;7:185-91. [published Online First: 2004/07/31]
6. Cooke M, Thackray S. Differences between community professional and patient perceptions of chronic obstructive pulmonary disease treatment outcomes: a qualitative study. *J Clin Nurs* 2012;21(11-12):1524-33. doi: 10.1111/j.1365-2702.2012.04094.x [published Online First: 2012/05/19]
7. Pagel C, Brown KL, McLeod I, et al. Selection by a panel of clinicians and family representatives of important early morbidities associated with paediatric cardiac surgery suitable for routine monitoring using the nominal group technique and a robust voting process. *BMJ open* 2017;7(5):e014743. doi: 10.1136/bmjopen-2016-014743 [published Online First: 2017/05/31]
8. Brown KL, Pagel C, Brimmell R, et al. Definition of important early morbidities related to paediatric cardiac surgery. *Cardiol Young* 2017;27(4):747-56. doi: 10.1017/S1047951116001256 [published Online First: 2016/09/30]
9. Rogers L, Brown KL, Franklin RC, et al. Improving Risk Adjustment for Mortality After Pediatric Cardiac Surgery: The UK PRAiS2 Model. *Ann Thorac Surg* 2017;104(1):211-19. doi: 10.1016/j.athoracsur.2016.12.014 [published Online First: 2017/03/21]
10. Varni JW, Seid M, Rode CA. The PedsQL: measurement model for the pediatric quality of life inventory. *Med Care* 1999;37(2):126-39. [published Online First: 1999/02/19]
11. Varni JW, Seid M, Kurtin PS. PedsQL 4.0: reliability and validity of the Pediatric Quality of Life Inventory version 4.0 generic core scales in healthy and patient populations. *Med Care* 2001;39(8):800-12. [published Online First: 2001/07/27]
12. Varni JW, Limbers CA, Neighbors K, et al. The PedsQL Infant Scales: feasibility, internal consistency reliability, and validity in healthy and ill infants. *Qual Life Res* 2011;20(1):45-55. doi: 10.1007/s11136-010-9730-5 [published Online First: 2010/08/24]
13. Kroenke K, Spitzer RL, Williams JB, et al. An ultra-brief screening scale for anxiety and depression: the PHQ-4. *Psychosomatics* 2009;50(6):613-21. doi: 10.1176/appi.psy.50.6.613 [published Online First: 2009/12/10]
14. Lowe B, Wahl I, Rose M, et al. A 4-item measure of depression and anxiety: validation and standardization of the Patient Health Questionnaire-4 (PHQ-4) in the general population. *Journal of affective disorders* 2010;122(1-2):86-95. doi: 10.1016/j.jad.2009.06.019 [published Online First: 2009/07/21]

For peer review only - http://bmjopen.bmj.com/site/about/guidelines.xhtml

15. https://www.picanet.org.uk/wp-content/uploads/sites/25/2018/05/PICANet_National_Report_2007.pdf. PCCMDS Leeds: HQIP; 2018 [accessed Jan 5 2018.
16. Norman GR, Sloan JA, Wyrwich KW. Interpretation of changes in health-related quality of life: the remarkable universality of half a standard deviation. *Med Care* 2003;41(5):582-92. doi: 10.1097/01.MLR.0000062554.74615.4C [published Online First: 2003/04/30]
17. Brown KL, Rogers L, Barron DJ, et al. Incorporating Comorbidity Within Risk Adjustment for UK Pediatric Cardiac Surgery. *Ann Thorac Surg* 2017;104(1):220-26. doi: 10.1016/j.athoracsur.2016.12.013 [published Online First: 2017/03/21]
18. Rubin DB. Multiple Imputation for Nonresponse in Surveys. In: Wiley, ed. New York, 1987.
19. Barber J, Thompson S. Multiple regression of cost data: use of generalised linear models. *Journal of health services research & policy* 2004;9(4):197-204. doi: 10.1258/1355819042250249 [published Online First: 2004/10/29]
20. 2015. S. Stata Statistical Software: Release 14. . In: LP. S, ed. College Station, TX:, 2015.
21. Aharon AS, Drinkwater DC, Jr., Churchwell KB, et al. Extracorporeal membrane oxygenation in children after repair of congenital cardiac lesions. *Annals of Thoracic Surgery* 2001;72(6):2095-101; discussion 101-2.
22. Alsoufi B, Shen I, Karamlou T, et al. Extracorporeal life support in neonates, infants, and children after repair of congenital heart disease: modern era results in a single institution. *Annals of Thoracic Surgery* 2005;80(1):15-21; discussion 21.
23. Beiras-Fernandez A, Deutsch M-A, Kainzinger S, et al. Extracorporeal membrane oxygenation in 108 patients with low cardiac output - a single-center experience. *International Journal of Artificial Organs* 2011;34(4):365-73.
24. Chauhan S, Malik M, Malik V, et al. Extra corporeal membrane oxygenation after pediatric cardiac surgery: a 10 year experience. *Annals of Cardiac Anaesthesia* 2011;14(1):19-24.
25. Coskun KO, Coskun ST, Popov AF, et al. Extracorporeal life support in pediatric cardiac dysfunction. *Journal Of Cardiothoracic Surgery* 2010;5:112.
26. Bojan M, Gerelli S, Gioanni S, et al. Evaluation of a new tool for morbidity assessment in congenital cardiac surgery. *Ann Thorac Surg* 2011;92(6):2200-4. doi: 10.1016/j.athoracsur.2011.08.017 [published Online First: 2011/11/26]
27. Costello JM, Cooper DS, Jacobs JP, et al. Intermediate-term outcomes after paediatric cardiac extracorporeal membrane oxygenation--what is known (and unknown). *Cardiol Young* 2011;21 Suppl 2:118-23. doi: 10.1017/S1047951111001697 [published Online First: 2011/12/14]
28. Jacobs ML, O'Brien SM, Jacobs JP, et al. An empirically based tool for analyzing morbidity associated with operations for congenital heart disease. *J Thorac Cardiovasc Surg* 2012 doi: 10.1016/j.jtcvs.2012.06.029 [published Online First: 2012/07/28]
29. Salvin JW, Scheurer MA, Laussen PC, et al. Blood transfusion after pediatric cardiac surgery is associated with prolonged hospital stay. *Ann Thorac Surg* 2011;91(1):204-10. doi: 10.1016/j.athoracsur.2010.07.037 [published Online First: 2010/12/22]
30. Utley M, Brown K, Tsang V. Invited commentary. *Ann Thorac Surg* 2012;94(5):1602-3. doi: 10.1016/j.athoracsur.2012.07.003 [published Online First: 2012/10/27]
31. Alten JA, Rahman AF, Zaccagni HJ, et al. The Epidemiology of Health-Care Associated Infections in Pediatric Cardiac Intensive Care Units. *Pediatr Infect Dis J* 2017 doi: 10.1097/INF.0000000000001884 [published Online First: 2017/12/28]
32. Hickey PA, Connor JA, Cherian KM, et al. International quality improvement initiatives. *Cardiol Young* 2017;27(S6):S61-S68. doi: 10.1017/S1047951117002633 [published Online First: 2017/12/05]
33. Gaies M, Werho DK, Zhang W, et al. Duration of Postoperative Mechanical Ventilation as a Quality Metric for Pediatric Cardiac Surgical Programs. *Ann Thorac Surg* 2018;105(2):615-21. doi: 10.1016/j.athoracsur.2017.06.027 [published Online First: 2017/10/11]

For peer review only - http://bmjopen.bmj.com/site/about/guidelines.xhtml

34. Buckley JR, Graham EM, Gaies M, et al. Clinical epidemiology and centre variation in chylothorax rates after cardiac surgery in children: a report from the Pediatric Cardiac Critical Care Consortium. *Cardiol Young* 2017:1-8. doi: 10.1017/S104795111700097X [published Online First: 2017/05/30]
35. Marino BS, Lipkin PH, Newburger JW, et al. Neurodevelopmental outcomes in children with congenital heart disease: evaluation and management: a scientific statement from the American Heart Association. *Circulation* 2012;126(9):1143-72.
36. Kwiatkowski DM, Goldstein SL, Cooper DS, et al. Peritoneal Dialysis vs Furosemide for Prevention of Fluid Overload in Infants After Cardiac Surgery: A Randomized Clinical Trial. *JAMA pediatrics* 2017;171(4):357-64. doi: 10.1001/jamapediatrics.2016.4538 [published Online First: 2017/02/28]

For peer review only

For peer review only - http://bmjopen.bmj.com/site/about/guidelines.xhtml

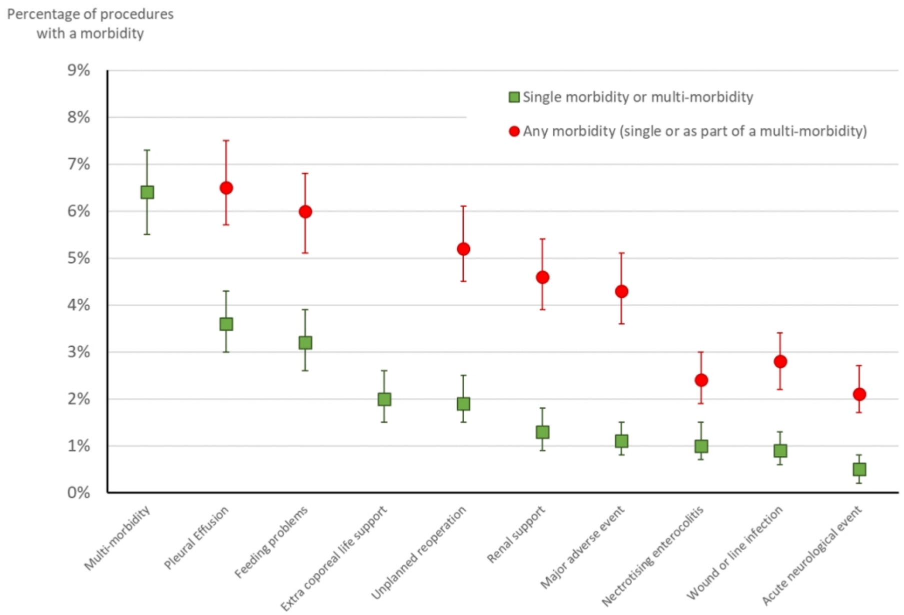

Figure 1 Incidence of morbidities
The figure shows the distinction between occurrence as a single morbidity and occurrence in combination with other morbidities, with 95% confidence intervals.

Appendix 1 from Brown KL, Pagel C, Brimmell R*, et al.* Definition of important early morbidities related to paediatric cardiac surgery. *Cardiol Young*. 2017;**27**:747-56 doi: 10.1017/S1047951116001256 [published Online First: 2016/09/30].

Morbidity	Timescale for identification	Definition	Measurement protocol (if additional to definition)	Minimum treatment protocol
**Acute Neurological Event**	Includes neurological morbidities that, based on best clinical judgement, arose as *new* findings around the time of surgery that were detected within the same hospitalisation as the surgery. It is recognised that in certain circumstances such as where a child is very sick on life support, pre procedure assessment is challenging, in these circumstances as full an evaluation as possible to be completed, incorporating serial assessments over time.	Neurological events including: seizure, abnormal movement (includes choreiform or athetoid), focal neurological deficit (includes hemiplegia and monoplegia), intracranial haemorrhage, stroke, brain death, Reversible Ischaemic Neurological Dysfunction, Hypoxic Ischaemic Encephalopathy, spinal cord ischaemia, basal ganglia damage or brain stem injury (includes abnormal cough or gag reflex). (1)	Includes new abnormality in any of the following:<br><br>- Electroencephalogram.<br><br>- Brain scan (either computerised tomography or magnetic resonance).<br><br>- Clinical evaluation<br><br>(Seizures or movement disorder, focal neurological signs, generalised neurological signs, altered conscious level including even brain death.)	The treatment protocol is variable depending on the type of neuro-morbidity.<br><br>Specialist consultation with a neurologist, a full evaluation of any brain injury and neuro-developmental follow up would be a minimum.

For peer review only - http://bmjopen.bmj.com/site/about/guidelines.xhtml

**Unplanned reoperation or re-intervention**	Unplanned re-interventions are procedures outside the expected patient pathway, which may be undertaken at any time from the start of the post-operative admission up until 30 days following the primary operation. Additional procedures or revisions undertaken within the primary trip to the operating theatre (incorporating return onto cardiopulmonary bypass) are not included in the definition of re-operation.	Unplanned re-interventions include procedures that were not intended during the planning phase, follow an initial primary cardiac surgery and result in "substantive alteration to heart" incorporating cardiac bypass, cardiac non bypass, pacemaker placement, interventional catheterisations and also diaphragm plications (which are not related to the heart itself). The definition does not include support or other non-cardiac surgery procedures.	Unplanned return to the operating room or cardiac catheter laboratory within 30 days<br><br>(Excludes interventional catheters that were planned pre-operatively; excluding delayed chest closure, excluding procedures for bleeding)<br><br>(Includes diaphragm plication and insertion of pace maker for surgically acquired arrhythmia).	Not applicable. The minimal assessment is cardiovascular evaluation of the repair with echocardiography and tolerance of weaning from life supports.
**Feeding problems**	A diagnosis of post-operative feeding problems should be considered during recovery after surgery and prior to discharge from the specialist centre either to home or to secondary care if	A child may fail to feed normally following paediatric cardiac surgery for a range of reasons including gastro-oesophageal reflux, vocal cord paralysis, oral-motor dysfunction, oral aversion, and neurologic impairment. (2) If for any of these reasons a child is not able to orally	The requirement for any feeding support.<br><br>Includes via the intravenous route or via an enteral tube.	Treatment includes assessment by the dietician, speech and language therapist and of the patient's weight. Progress with

For peer review only - http://bmjopen.bmj.com/site/about/guidelines.xhtml

	the child is unable to feed normally. The goal is detection of feeding problems which are new post-surgery, and it is recognised that this may be challenging where a child was not fed pre-operatively for cardiac reasons since feeding ability will not have been assessed objectively.	feed or completely orally feed and is tube dependent at discharge from the tertiary centre or at 30 days (if he or she is otherwise clinically stable enough to feed at that time point), then a post-operative feeding problem will be diagnosed.	Excludes feeding support that was present to treat a primary problem diagnosed before the surgery, feeding support related to an episode of necrotising enterocolitis, and feeding support because the child dislikes a special diet.	feeding should be monitored by the clinical care team responsible at each stage of the journey.
**Need for renal replacement therapy**	Includes renal replacement therapy when initiated as a new support at any time from the start of the post-operative admission to ICU up until 30 days following the primary operation.	The child requires renal replacement therapy (peritoneal dialysis or haemofiltration) for renal failure (oligo-anuria of less than 0.5 ml/kg/hour and elevated creatinine level for age) and or fluid overload. In patients where renal support is required alongside extracorporeal life support, the primary morbidity is viewed as extracorporeal life support.	The measurement protocol is simply the presence of (new) renal support. (Excludes renal support on extracorporeal life support). Data on renal biochemistry and urine output will be collected.	Instigation of effective renal replacement therapy.<br><br>If recovery of kidney function does not occur within 3 to 5 weeks then consultation with paediatric renal

For peer review only

				physician is required.
**Major adverse cardiac events or never events**	Events within this morbidity may be identified during the tertiary hospital stay (either ward or ICU) following the primary surgery.	These morbidity includes:<br><br>- Cardiac arrest, where the child receives any chest compressions or defibrillation.<br><br>- Chest re-opening on the ICU or ward for any reason.<br><br>- Major haemorrhage in the ICU following surgery.<br><br>- A 'Never Event' applicable to paediatric cardiac surgery as selected from the 'Never Events' list published for NHS for 2015 (3)<br><br>(Including wrong site or wrong patient surgery, wrong prosthesis surgery, retained foreign object post procedure, wrong route administration of medication, transfusion or transplantation of main red cell group incompatible blood components or organs, misplaced naso-gastric or oro-gastric tubes,	Major haemorrhage is defined as bleeding > 10ml/kg/hr on ICU for 2 consecutive hours.<br><br>A 'Never Event' includes the events listed plus harm to the patient, for example: if a naso-gastric tube is misplaced, detected and removed in a timely manner before any harm is done then this is not a 'Never Event'. Conversely, if the misplaced naso-gastric tube is not noted, and feed is given into the bronchus, then this is a 'Never Event'.	All events will results in immediate treatment as part of current practice.

For peer review only

		- Tissue injury to limb or vital organ such as perforated viscus or ischaemic limb injury.		
**Extracorporeal life support**	Extracorporeal life support following surgery and before discharge from the tertiary hospital, including the rare cases when a child was on extracorporeal life support before surgery.	This morbidity is defined by the presence of an extracorporeal life support system connected to the patient following the operation, whether it was placed in the operating theatre or in the intensive care unit, and whether the indication was cardiac arrest, low cardiac output state, poor cardiac function, arrhythmia, residual or recurrent cardiac lesion, pulmonary including pulmonary hypertension or sepsis.	It is recognised that children on extracorporeal life support following paediatric cardiac surgery have high rates of other complications including renal support, bleeding, sepsis, sternal reopening, and cardiac arrest. (4) Where such complications arise as part of extracorporeal life support, the morbidity is defined as extracorporeal life support.	The morbidity is in fact a treatment modality offered so this is not applicable. Centres offering extracorporeal life support follow protocols based on those provided by the extracorporeal life support organisation. [24]
**Necrotising enterocolitis**	Necrotising enterocolitis as a new diagnosis from after surgery until discharge from the tertiary hospital.	Necrotising enterocolitis class 1a or 1b, (5) which incorporates babies with systemic signs of inflammation and abdominal clinical signs such as distension or larger than normal gastric aspirates or mild rectal	Data in respect of systemic clinical signs, intestinal signs and radiology will be collected, as well as the	Consultation with general surgery and further management in respect of antibiotics,

For peer review only

		bleeding but no radiological changes are included, if a general surgery specialist has seen the child and commenced a course of intravenous antibiotics and parenteral nutrition for five to seven days. Cases of severe necrotising enterocolitis with radiological signs systemic instability and bowel perforation are also included.	treatments deployed, thus enabling the necrotising enterocolitis diagnosis to be graded between 1a and 3b. [25]	nutrition, radiological investigation and surgical intervention.
**Surgical site infection and bloodstream infection**	Surgical site and blood stream infections diagnosed within the hospital admission following surgery or following readmission to the same unit during post-operative recovery, where the treating clinical team assesses the infection to be linked to the recent operation. It is noted that mediastinitis may be detected more than 30 days after cardiac surgery (6) hence this time cut off is not applicable.	Deep surgical site Infection and/or mediastinitis includes any infection of an incised wound that undergoes any re intervention by a surgeon (such as opening of the wound, vacuum dressing), mediastinitis and false aneurysm, independent of culture positivity. [23]<br><br>Blood stream infection includes both catheter related and non-catheter related. Cases have systemic signs of infection, a positive culture not judged to be a contaminant, and in the case of line related a catheter in place with positive cultures from the line or from the line tip when removed.	Deep surgical site infection excludes superficial site infection managed without a surgeon's reoperation by conventional nurse dressing only, even if the wound heals by secondary intention.	The minimum treatment protocol consists of antibiotics based on organism and sensitivities, and where relevant the removal of the line. Surgical intervention may be required for deep surgical site and in some cases of endocarditis. Both conditions require

For peer review only

		Endocarditis based on clinical, imaging or culture evidence judged to be diagnostic of endothelial/endocardial infection and its sequelae cardiac or extra-cardiac.		prolonged antibiotic therapy.
**Prolonged pleural effusion or chylothorax**	Prolonged pleural effusion is a post procedural effusion with duration greater than ten days. Chylothorax is diagnosed from after surgery until discharge from the tertiary hospital.	Either a chylous pleural effusion or significant chylous pericardial effusion or significant chylous ascites or a prolonged non-chylous effusion that necessitates thoracic drainage at least ten days following index cardiac surgery.	Chylous effusions are characterised by milky appearance and a pleural fluid white blood cell count of greater than 1000 cells/μl with lymphocytes greater than 80%. (7) If the child is on normal feeds the triglyceride level in the pleural fluid will be > 1.1 mmol/L or the ratio between the pleural triglyceride level and the serum triglyceride level will exceed 1.	Diet consisting of medium chain triglycerides or low fat for chylothorax. On a patient-by-patient basis other treatments include parenteral nutrition, octreotide infusion, intervention for venous obstruction thoracic duct ligation, and pleuradhesis.

For peer review only

1. Dominguez TE, Wernovsky G, Gaynor JW. Cause and prevention of central nervous system injury in neonates undergoing cardiac surgery. *Seminars in thoracic and cardiovascular surgery*. 2007;**19**:269-77 doi: 10.1053/j.semtcvs.2007.07.005 [published Online First: 2007/11/07].
2. Medoff-Cooper B, Naim M, Torowicz D, Mott A. Feeding, growth, and nutrition in children with congenitally malformed hearts. *Cardiol Young*. 2010;**20 Suppl 3**:149-53 doi: 10.1017/S1047951110001228 [published Online First: 2010/11/20].
3. NHS. Never-events. In: England N, ed. *Patient Safety*. London: NHS England 2015:Patient Safety Guideline.
4. Chaturvedi RR, Macrae D, Brown KL, *et al*. Cardiac ECMO for biventricular hearts after paediatric open heart surgery. *Heart*. 2004;**90**:545-51 Online First: 2004/04/16].
5. McElhinney DB, Hedrick HL, Bush DM, *et al*. Necrotizing enterocolitis in neonates with congenital heart disease: risk factors and outcomes. *Pediatrics*. 2000;**106**:1080-7 Online First: 2000/11/04].
6. Sohn AH, Schwartz JM, Yang KY, Jarvis WR, Guglielmo BJ, Weintrub PS. Risk factors and risk adjustment for surgical site infections in pediatric cardiothoracic surgery patients. *Am J Infect Control*. 2010;**38**:706-10 doi: 10.1016/j.ajic.2010.03.009 [published Online First: 2010/07/08].
7. Zuluaga MT. Chylothorax after surgery for congenital heart disease. *Curr Opin Pediatr*. 2012;**24**:291-4 doi: 10.1097/MOP.0b013e3283534b7f [published Online First: 2012/04/14].

For peer review only

For peer review only

Pages Based on R2 marked word version.

STROBE Statement—checklist of items that should be included in reports of observational studies

	Item No	Recommendation	
**Title and abstract**	1	(*a*) Indicate the study's design with a commonly used term in the title or the abstract	(1)
		(*b*) Provide in the abstract an informative and balanced summary of what was done and what was found	(2-3)
**Introduction**			
Background/rationale	2	Explain the scientific background and rationale for the investigation being reported	(6)
Objectives	3	State specific objectives, including any prespecified hypotheses	(6)
**Methods**			
Study design	4	Present key elements of study design early in the paper	(6-7)
Setting	5	Describe the setting, locations, and relevant dates, including periods of recruitment, exposure, follow-up, and data collection	(7-8)
Participants	6	(*a*) *Cohort study*—Give the eligibility criteria, and the sources and methods of selection of participants. Describe methods of follow-up<br>*Case-control study*—Give the eligibility criteria, and the sources and methods of case ascertainment and control selection. Give the rationale for the choice of cases and controls<br>*Cross-sectional study*—Give the eligibility criteria, and the sources and methods of selection of participants	(7-8)
		(*b*) *Cohort study*—For matched studies, give matching criteria and number of exposed and unexposed<br>*Case-control study*—For matched studies, give matching criteria and the number of controls per case	8
Variables	7	Clearly define all outcomes, exposures, predictors, potential confounders, and effect modifiers. Give diagnostic criteria, if applicable	(7-10)
Data sources/ measurement	8*	For each variable of interest, give sources of data and details of methods of assessment (measurement). Describe comparability of assessment methods if there is more than one group	(8-10)
Bias	9	Describe any efforts to address potential sources of bias	
Study size	10	Explain how the study size was arrived at	9
Quantitative variables	11	Explain how quantitative variables were handled in the analyses. If applicable, describe which groupings were chosen and why	(10-11)
Statistical methods	12	(*a*) Describe all statistical methods, including those used to control for confounding	(10-11)
		(*b*) Describe any methods used to examine subgroups and interactions	(10-11)
		(*c*) Explain how missing data were addressed	(10-11)
		(*d*) *Cohort study*—If applicable, explain how loss to follow-up was addressed<br>*Case-control study*—If applicable, explain how matching of cases and controls was addressed<br>*Cross-sectional study*—If applicable, describe analytical methods taking account of sampling strategy	10<br>(7+8)<br>(10-11)
		(*e*) Describe any sensitivity analyses	11

Continued on next page

For peer review only - http://bmjopen.bmj.com/site/about/guidelines.xhtml

**Results**			
Participants	13*	(a) Report numbers of individuals at each stage of study—eg numbers potentially eligible, examined for eligibility, confirmed eligible, included in the study, completing follow-up, and analysed	(P 12 and 14)
		(b) Give reasons for non-participation at each stage	P 14
		(c) Consider use of a flow diagram	N/A
Descriptive data	14*	(a) Give characteristics of study participants (eg demographic, clinical, social) and information on exposures and potential confounders	Tab1
		(b) Indicate number of participants with missing data for each variable of interest	(Tabs 1, 2, 3, 4)
		(c) *Cohort study*—Summarise follow-up time (eg, average and total amount)	(Tab 2, p14)
Outcome data	15*	*Cohort study*—Report numbers of outcome events or summary measures over time	N/A
		Case-control study—Report numbers in each exposure category, or summary measures of exposure	[illegible]
		Cross-sectional study—Report numbers of outcome events or summary measures	Tab1
Main results	16	(*a*) Give unadjusted estimates and, if applicable, confounder-adjusted estimates and their precision (eg, 95% confidence interval). Make clear which confounders were adjusted for and why they were included	Tab1
		(*b*) Report category boundaries when continuous variables were categorized	Tab1
		(*c*) If relevant, consider translating estimates of relative risk into absolute risk for a meaningful time period	N/A
Other analyses	17	Report other analyses done—eg analyses of subgroups and interactions, and sensitivity analyses	(Tabs 3, 4)
**Discussion**			
Key results	18	Summarise key results with reference to study objectives	(p 17 – 18)
Limitations	19	Discuss limitations of the study, taking into account sources of potential bias or imprecision. Discuss both direction and magnitude of any potential bias	P 18
Interpretation	20	Give a cautious overall interpretation of results considering objectives, limitations, multiplicity of analyses, results from similar studies, and other relevant evidence	(p 18 – 19)
Generalisability	21	Discuss the generalisability (external validity) of the study results	(P 18 – 19)
**Other information**			
Funding	22	Give the source of funding and the role of the funders for the present study and, if applicable, for the original study on which the present article is based	P5.

*Give information separately for cases and controls in case-control studies and, if applicable, for exposed and unexposed groups in cohort and cross-sectional studies.

Note: An Explanation and Elaboration article discusses each checklist item and gives methodological background and published examples of transparent reporting. The STROBE checklist is best used in conjunction with this article (freely available on the Web sites of PLoS Medicine at http://www.plosmedicine.org/, Annals of Internal Medicine at http://www.annals.org/, and Epidemiology at http://www.epidem.com/). Information on the STROBE Initiative is available at www.strobe-statement.org.
